# Impaired Interneuron Development in a Novel Model of Neonatal Brain Injury

**DOI:** 10.1523/ENEURO.0300-18.2019

**Published:** 2019-02-22

**Authors:** Helene Lacaille, Claire-Marie Vacher, Dana Bakalar, Jiaqi J. O’Reilly, Jacquelyn Salzbank, Anna A. Penn

**Affiliations:** 1 Center for Neuroscience, Children’s National Health System, Washington, DC 20010; 2 Institute for Biomedical Sciences, George Washington University, Washington, DC 20037; 3 Fetal Medicine Institute, Neonatology, Children’s National Health System, Washington, DC 20010

**Keywords:** hypoxia, inflammation, interneurons, prematurity, psychiatric disorders

## Abstract

Prematurity is associated with significantly increased risk of neurobehavioral pathologies, including autism and schizophrenia. A common feature of these psychiatric disorders is prefrontal cortex (PFC) inhibitory circuit disruption due to GABAergic interneuron alteration. Cortical interneurons are generated and migrate throughout late gestation and early infancy, making them highly susceptible to perinatal insults such as preterm birth. Term and preterm PFC pathology specimens were assessed using immunohistochemical markers for interneurons. Based on the changes seen, a new preterm encephalopathy mouse model was developed to produce similar PFC interneuron loss. Maternal immune activation (MIA; modeling chorioamnionitis, associated with 85% of extremely preterm births) was combined with chronic sublethal hypoxia (CSH; modeling preterm respiratory failure), with offspring of both sexes assessed anatomically, molecularly and neurobehaviorally. In the PFC examined from the human preterm samples compared to matched term samples at corrected age, a decrease in somatostatin (SST) and calbindin (CLB) interneurons was seen in upper cortical layers. This pattern of interneuron loss in upper cortical layers was mimicked in the mouse PFC following the combination of MIA and CSH, but not after either insult alone. This persistent interneuron loss is associated with postnatal microglial activation that occurs during CSH only after MIA. The combined insults lead to long-term neurobehavioral deficits which parallel human psychopathologies that may be seen after extremely preterm birth. This new preclinical model supports a paradigm in which specific cellular alterations seen in preterm encephalopathy can be linked with a risk of neuropsychiatric sequela. Specific interneuron subtypes may provide therapeutic targets to prevent or ameliorate these neurodevelopmental risks.

## Significance Statement

Growing evidence suggests that a common component of psychiatric disorders is damage to inhibitory neurons. In the frontal lobe, these neurons continue to develop during late gestation and infancy, predisposing preterm survivors to neurobehavioral disorders and/or cognitive impairment. Preventing neuronal damage depends on having accurate models of preterm brain injury with well-defined outcome measures that can be examined in both small animals and humans. In this study, interneuron number were assessed in term and preterm human frontal brain tissues and in a novel multifactorial model that combines prenatal inflammation with postnatal hypoxia resulting in long-term inhibitory neuron loss. This new model was validated through anatomic and functional assessments that directly translate to human measures.

## Introduction

Dysfunction of the prefrontal cortex (PFC) underlies a number of deficits associated with psychiatric disorders, particularly their cognitive and social components (Sugranyes et al., 2011). A common feature of these psychiatric disorders is a disruption of PFC inhibitory circuits, mediated by developmental alterations of GABA interneurons (Marin, 2012). Interneuron loss, misplacement, and dysmaturation characterize these disorders (Gogolla et al., 2009; Meechan et al., 2009; Takano, 2015; Filice et al., 2016; Hashemi et al., 2017). Specifically, GABAergic network alteration in the Brodmann area 9 (BA9; dorsolateral PFC), a region involved in working memory and social cognition, may account for specific cognitive deficits of these disorders (Tooney and Chahl, 2004; Samaco et al., 2005; Fatemi et al., 2009; Habl et al., 2012; Hashemi et al., 2017).

GABA progenitor cells proliferate in the fetal brain (at least through 35 weeks of gestation; Arshad et al., 2016) and then migrate to the frontal lobes and mature during infancy (Paredes et al., 2016), leaving these interneurons precursors highly susceptible to perinatal insults. A correlation between prematurity, GABA concentration and abnormal functional connectivity has been demonstrated in the BA9 (Kwon et al., 2014), raising the possibility that alterations in interneuron number or function may contribute to increase the risk of later neurodevelopmental psychopathology associated with preterm birth (Johnson and Marlow, 2011).

Preterm birth (delivery before 37 weeks of gestation) is a multifactorial syndrome. Chorioamnionitis occurs in up to 85% of extremely preterm births and is strongly associated with an increased risk of spontaneous preterm delivery (Blencowe et al., 2013). After preterm birth, additional insults such as respiratory failure can increase the risk of long-term impairments, including disorders of executive functioning, autism, and schizophrenia (Adams-Chapman and Stoll, 2006; Arpino et al., 2010; Meldrum et al., 2013; Knuesel et al., 2014; Lombardo et al., 2017).

Understanding interneuron alterations after preterm birth can contribute to a better comprehension of the perinatal environment’s impact on psychiatric disorders. In this study, examination of a set of human preterm and term postmortem samples demonstrated decreased density of specific PFC interneuron subpopulations. To investigate this finding of persistent interneuron loss, a novel multi-insult mouse model was then developed, using both prenatal maternal immune activation (MIA) and postnatal chronic sublethal hypoxia (CSH), designed to capture the type and timing of common perinatal physiologic insults. Prior models have induced early MIA resulting in limited deficits of cortical interneurons (Carpentier et al., 2013; Canetta et al., 2016). Likewise, postnatal insults during the first two weeks of rodent life, such as hypoxia-ischemia or CSH, have been used to mimic white matter abnormalities that develop in preterm infants (Mayoral et al., 2009; Salmaso et al., 2014; Scafidi et al., 2014; Zonouzi et al., 2015). Limited alterations of interneuron numbers (Fagel et al., 2009) or delayed interneuron maturation (Komitova et al., 2013) have been described after these postnatal insults, but these changes do not recapitulate the human pathology depicted here. Rather, unlike embryonic MIA or postnatal CSH alone, the combination of insults significantly disrupts the density of mature interneurons in the PFC, mimicking the cellular alterations seen in the samples from extremely preterm neonates examined here- and induces specific cognitive deficits.

This multi-hit protocol is proposed as a novel preclinical model to decipher the cellular and physiologic mechanisms underlying the deficits seen in preterm survivors exposed to inflammation and hypoxia that may make them vulnerable to later neuropsychiatric disorders.

## Materials and Methods

### Study approval

All animal procedures were performed in accordance with the Children’s National Institutional Animal Care and Use Committee (#00030312).

### Human samples

Human samples were obtained from the NIH NeuroBioBank at University of Maryland, Baltimore, MD (ID #709). Donors consisted of corrected age preterm and matched term infants, excluding those with major congenital anomalies, or known genetic diagnoses and those with meningitis or stroke as cause of death. Sex, gestational weeks, absolute age (postnatal age), and corrected age are shown in Table 1. BA9 formalin-fixed brain samples were cut into 0.5-cm-thick coronal slices and preserved in 10% neutral buffered formalin; matched frozen tissues were preserved at –80°C.

**Table 1.** List of donors (from NIH NeuroBioBank, University of Maryland, Baltimore, MD)

Preterm infants					
Accession number	Sex	Gestational weeks	Absolute age (months)	Corrected age (months)	Cause of death
–1224	M	31	1.3	–1	Sudden unexplained death in infancy
437	M	28	3.3	0.3	Sudden unexplained death in infancy
934	M	27	4.5	1.3	Sudden unexplained death in infancy
1325	F	25	6.1	2.3	Sudden unexplained death in infancy
1487	F	29	2.1	–0.6	Prematurity with complications
4364	M	27	4.8	1.6	Prematurity, pneumonia
4373	F	34	3.3	1.8	Methicillin susceptible *Staphylococcus*
4389	F	34	2.6	1.1	Positional asphyxia
4416	F	26	4.7	1.2	Asphyxia, prematurity
4417	M	28	2.4	–0.6	Undetermined, hepatic stenosis, prematurity
5708	F	29	5.2	2.5	Viral syndrome with focal acute pneumonia
5716	M	29	3.2	0.5	Sudden unexplained death in infancy
5754	M	33	5	3.2	Sudden unexplained death in infancy
5843	M	34	3.3	1.8	Sudden unexplained death in infancy
Term control infants					
Accession number	Sex	Gestational weeks	Absolute age (months)	Corrected age (months)	Cause of death
4353	M	40	1.1	1.1	Positional asphyxia
4355	M	38	2.7	2.2	Prone sleep position and excessive bedding
4375	F	40	0.1	0.1	Positional asphyxia
4381	F	40	3	3	Probable asphyxia
4383	F	40	2.5	2.5	Probable overlay
4391	M	40	0.9	0.9	Asphyxia due to co-sleeping
4402	M	39	2.2	2	Co-sleeping
4412	M	40	2.2	2.2	Sudden unexplained infant death
4414	F	37	1.3	0.5	Sudden unexpected infant death with co-sleeping
4420	M	40	2.1	2.1	Positional asphyxia
5658	M	38	1.4	0.9	Sudden unexplained death in infancy
5761	F	38	1.1	0.6	Sudden unexplained death in infancy
5886	M	40	1.5	1.5	Sudden unexplained death in infancy

### Animals

Experimental mice were produced by a heterozygous breeding scheme in which *GAD65-GFP* transgenics (C57BL/6 background; obtained from Dr. Vittorio Gallo, generated from Dr. Gabor Szabo; López-Bendito et al., 2004) were crossed to C57BL/6 mice. For time pregnant mating, male and female pairs were housed overnight, with the following day designated as embryonic day (E)0.5. The day of birth was designated as postnatal day (P)0. For all experiments described here, embryos and postnatal pups of both sexes were included. Each experimental group contained pups from at least two litters.

### MIA and CSH

Mild, late MIA was induced using 150 µg/kg of lipopolysaccharide (LPS; L6529, from *Escherichia coli* O55:B5, Millipore Sigma), administered to the pregnant dam intraperitoneally on both E15.5 and E16.5. After delivery, dams and litters were housed in 10.9% oxygen from P3 to P10 to produce CSH, with control litters housed in the same room outside of the hypoxia chamber. No adverse effects of hypoxia were noted on dams or pups, except for mild growth restriction that resolved over the first postnatal month (Salmaso et al., 2015). Four experimental groups were studied: mice treated with saline and reared under normoxia (MIA–/CSH–), mice subjected to MIA and reared under normoxia (MIA+/CSH–), mice treated with saline and reared under CSH (MIA–/CSH+), and mice subjected to MIA and reared under CSH (MIA+/CSH+). Mice from multiple litters were randomly assigned to these groups and groups were balanced for litter size and sex.

### Immunohistochemical procedure

#### Human tissue

Formalin-fixed tissues were cryoprotected in a 30% sucrose solution and embedded in Tissue-Tek O.C.T. Compound (Sakura Finetek). Blocks were cut into 25-µm-thick sections on a cryostat and mounted on Superfrost Plus (ThermoFisher) glass slides. Frozen sections were allowed to equilibrate to room temperature for 2 h before staining.

#### Mouse tissue

Embryonic brains were obtained after euthanasia of the dam by CO_2_ asphyxiation followed by cerebral dislocation; brains were quickly dissected in 1× PBS and transferred to 4% paraformaldehyde (PFA). Postnatal mice were perfused at P10 or P30 with 1× PBS/4% PFA, and brains were postfixed in 4% PFA for 24 h and transferred into 30% sucrose in 1× PBS. Brains were sectioned into coronal 40-μm-thick sections with a sliding microtome before immunolabeling.

#### Procedure

Tissue sections were rinsed in PBS-Triton X-100 0.3% (PBS-T) then blocked in PBS-T with 10% normal donkey serum (NDS) followed by overnight incubation at 4°C in PBS-T-10% NDS with primary antibodies: BrdU (1:500, Abcam, ab6326), calbindin (CLB; 1:1000, Swant Marly, Cb300 or Cb38), calretinin (CRT; 1:1000, Millipore Sigma AB1550), cleaved-caspase 3 (1:500, Cell Signaling Technology, Asp175), CD68 (1:300, Bio-Rad, MCA1957GA), Gad65-67 (1:200 Santa Cruz Biotechnology sc-365180), Gad67 (1:100, Millipore Sigma MAB5406), GFP (1:500, Abcam, Ab13970), Iba1 (1:500, Wako Chemicals, 019-19741), Ki67 (1:500, Abcam, ab15580), NeuN (1:500, Abcam, ab177487), neuropetide Y (1:500, Immunostar, 22940), parvalbumin (PV; 1:1000, Millipore Sigma, P3088), somatostatin (SST; 1:300, Santa Cruz Biotechnology, sc7819), reelin (RLN; 1:300, R&D Systems, AF3820), and VIP (1:1000, Immunostar, 20077). For secondary detection, appropriately matched Alexa Fluor-conjugated secondary antibodies (1:500, ThermoFisher) were incubated 90 min in PBS-T at room temperature. For all groups studied, dividing cells were detected with bromodeoxyuridine (BrdU, Roche) injected at E15.5 intraperitoneally at 50 mg/kg. For BrdU staining, a DNA denaturation step was performed by incubating the sections in 2 N HCl for 30 min at 45°C before the primary antibody incubation. Sections were incubated with DAPI, mounted in Fluoromount G (ThermoFisher) and coverslipped before confocal examination (Olympus FV1000).

#### Quantification

For human sections, the cell density was assessed in the upper layers (ULs), lower layers (LLs), and the subcortical white matter (SWM) of BA9 and expressed in cells/mm^2^. Cortical layering was determined with DAPI counterstaining. For mouse tissues, results from four sections per animal and five to six animals per group were average and the number of cells in each cortical sub-region was expressed as density of cells/mm^3^. Cortical layering was determined with DAPI counterstaining (Skorput et al., 2015). The delineation between the anterior cingulate cortex (ACC) and the prelimbic area (PL) is based on the Allen Brain Atlas. Cell quantification was performed using the Imaris-Bitplane software. All counts were performed blind to condition.

### Real-time PCR (RT-PCR)

#### Human tissue

BA9 tissues were homogenized in TRIzol Reagent (ThermoFisher, 15596018); total RNA was extracted with the RNeasy Mini kit (QIAGEN, 74104).

#### Mouse tissue

Cerebral cortices were collected in RNA later (QIAGEN, 76106); total RNA was extracted with the PARIS kit (ThermoFisher, am1921) and quantified with a Nanodrop ND-2000C (ThermoFisher).

#### Procedure

A total of 1 µg of RNA was used to make cDNA with the iScript cDNA Synthesis kit (Bio-Rad, 1708891). All primer pairs were designed and validated in-house for efficiency and specificity. RT-PCR experiments were performed on cDNA samples in presence of SsoAdvanced Universal SYBR Green Supermix (Bio-Rad, 1725271) with specific primers at 100 nM using the ABI Prism 7500 Sequence Detection System, ThermoFisher). The cDNA-generated signals for target genes were internally corrected with transferrin receptor protein 1 (*tfrc*) for human tissues and phosphoglycerate kinase 1 (*pgk1*) for mouse tissues. The regulation was determined with the 2^-ΔΔCq^ method.

### Western blotting

Human samples were homogenized in RIPA lysis buffer with proteinase inhibitors (Santa Cruz Biotechnology, sc24948). Protein extracts, 40 μg per lane, were loaded onto 4–20% gradient gels (NuSep Inc, NB10-420). Gels were electrotransfered to a 0.2-μm nitrocellulose membrane (Bio-Rad, 1620174). Blots were blocked in 5% milk in TBST for 1 h, and then incubated at 4°C overnight with one of the following antibodies: anti-gad65; anti-gad67; anti-SST; anti-gapdh (Santa Cruz Biotechnology, sc377145, sc28376, sc7819, and sc32233, respectively), anti-CLB (Swant Marly, Cb38), and anti-CRT (Millipore Sigma, AB1550). Bands were detected with appropriate horseradish peroxide-conjugated secondary antibodies, reacted with chemiluminescent ECL substrate (Bio-Rad, 1705060) and visualized with a Bio-Rad ChemiDoc Imaging system. Band intensity was measured using the ImageJ program (NIH).

### Behavioral experiments

Testing began at P30 and a one-week intertest interval was provided between each test paradigm.

#### Barnes maze investigation and reinvestigation

The Barnes maze (Stoelting Co) consisted of a circular disk with equally spaced holes. The number and position of nose-pokes over a 10-min period were recorded for each animal. Repeated pokes into previously explored holes were divided by the number of initial pokes.

#### Y-maze spontaneous alternation

Mice were placed in a Y-shaped maze. The number of arm entries over a 10-min period was recorded. The alternation index was calculated as the number of total complete alternations, divided by the total number of arm entries minus two and expressed as a percentage.

#### Water T-maze and reversal learning

Testing occurred in a 6-cm-deep T-maze (Stoelting Co) filled with water (22 ± 1°C). A clear platform 10 mm under the water surface was placed at the end of the left or right arms. On days 1–5, animals were trained to locate the hidden platform with four trials per day. On day 6, the platform location was switched to the opposite arm of the T-maze and training continued until day 9.

#### Socialization test

Testing occurred in a three-chamber box (Stoelting Co). The two outer compartments contained metal wire cages where stranger mice 1 and 2 (S1 and S2) were held. After 10 min of habituation, the test animal was placed in the middle chamber with the adjacent doors closed. S1 was placed in one cage and an object (O) in the other. The doors were opened to allow the test animal 10 min exploration. Social interaction duration (test mouse nose in contact with cage containing S1) was measured. S2 subsequently replaced the O and the test mouse was allowed another 10 min exploration. The time spent with either S1 or S2 was divided by the time spent with S1+O for social preference, or S1+S2 for novelty, and expressed as percentage.

#### Open field

Anxiety and activity were examined in an open field (40 × 40 cm; Stoelting). Mice were recorded and analyzed over 10 min with ANY-maze (Stoelting). Tracing paths of the mice were recorded and time spent in the central part (25 × 25 cm) versus time spent at the border was evaluated.

#### Marble burying

This test for repetitive behavior was performed in a box filled with 5 cm of bedding. Twelve glass marbles were evenly placed on the surface of the bedding. The number of buried marbles (to 2/3 of their depth) was counted after a 30-min exploration period.

### Statistics

All experiments and analysis were performed blind to conditions. Statistical analysis was performed using PRISM software (GraphPad Software 6.0). Normal distribution of each dataset was analyzed by Shapiro–Wilk test. When two conditions were compared, data were analyzed with a nonparametric Mann–Whitney test. When four experimental groups were assessed, and three conditions compared to the control group, data were analyzed with a one-way ANOVA with Holm–Sidak’s multiple comparisons or Kruskal–Wallis with Dunn’s multiple comparisons. When four experimental groups were assessed and two variables were taken into consideration, data were analyzed with a two-way ANOVA with Fisher LSD, Sidak’s or Tukey’s multiple comparisons. In Figure 2, the statistical analysis was performed layer by layer with a Kruskal–Wallis with Dunn’s multiple comparisons. The null hypothesis was rejected for α > 5%. The Statistical analysis is reported in detail in Table 2.

**Table 2.** Statistical analysis

Dataset	Data structure	Type of test	Power
Fig. 1*A*	Non-normal distribution	Two-way ANOVA, uncorrected Fisher's LSD	UL: *p* = 0.1337, LL: *p* = 0.4334, SWM: *p* = 0.5985
Fig. 1*B*	Non-normal distribution	Two-way ANOVA, uncorrected Fisher's LSD	UL: *p* = 0.0425, LL: *p* = 0.9257, SWM: *p* = 0.9609
Fig. 1*C*	Non-normal distribution	Two-way ANOVA, uncorrected Fisher's LSD	UL: *p* = 0.0278, LL: *p* = 0.4931, SWM: *p* = 0.7801
Fig. 1*D*	Non-normal distribution	Two-way ANOVA, uncorrected Fisher's LSD	UL: *p* = 0.2880, LL: *p* = 0.6393, SWM: *p* = 0.0240
Fig. 1*E*	Non-normal distribution	Two-way ANOVA, uncorrected Fisher's LSD	UL: *p* = 0.9257, LL: *p* = 0.9397, SWM: *p* = 0.2848
Extended Data Fig. 1-1*A*	Non-normal distribution	Two-way ANOVA, uncorrected Fisher's LSD	UL: *p* = 0.9265, LL: *p* = 0.5300, SWM: *p* = 0.4023
Extended Data Fig. 1-1*B*	Non-normal distribution	Two-way ANOVA, uncorrected Fisher's LSD	UL: *p* = 0.8052, LL: *p* = 0.3294, SWM: *p* = 0.8217
Extended Data Fig. 1-1*C*	Non-normal distribution	Two-way ANOVA, uncorrected Fisher's LSD	UL: *p* = 0.3699, LL: *p* = 0.9644, SWM: *p* = 0.9559
Extended Data Fig. 1-1*D*	Non-normal distribution	Two-way ANOVA, uncorrected Fisher's LSD	UL: *p* = 0.3370, LL: *p* = 0.2271, SWM: *p* = 0.3900
Extended Data Fig. 1-1*E*	Non-normal distribution	Two-way ANOVA, uncorrected Fisher's LSD	UL: *p* = 0.7681, LL: *p* = 0.7659, SWM: *p* = 0.6875
Extended Data Fig. 1-2*B*	Non-normal distribution	Two-way ANOVA, Sidak's multiple comparisons	Male: *p* = 0.9428, female: *p* = 0.7868
Extended Data Fig. 1-2*C*	Non-normal distribution	Two-way ANOVA, Sidak's multiple comparisons	Male: *p* = 0.3033, female: *p* = 0.8720
Extended Data Fig. 1-2*D*	Non-normal distribution	Two-way ANOVA, Sidak's multiple comparisons	Male: *p* = 0.9960, female: *p* = 0.7304
Extended Data Fig. 1-2*E*	Non-normal distribution	Two-way ANOVA, Sidak's multiple comparisons	Male: *p* = 0.9940, female: *p* = 0.9212
Extended Data Fig. 1-2*F*	Non-normal distribution	Two-way ANOVA, Sidak's multiple comparisons	Male: *p* = 0.8677, female: *p* = 0.8035
Extended Data Fig. 1-3*A*	Non-normal distribution	Two-way ANOVA, Sidak's multiple comparisons	Male: *p* = 0.6754, female: *p* = 0.9993
Extended Data Fig. 1-3*B*	Non-normal distribution	Two-way ANOVA, Sidak's multiple comparisons	Male: *p* = 0.0497, female: *p* = 0.4501
Extended Data Fig. 1-3*C*	Non-normal distribution	Two-way ANOVA, Sidak's multiple comparisons	Male: *p* = 0.7528, female: *p* = 0.6561
Extended Data Fig. 1-3*D*	Non-normal distribution	Two-way ANOVA, Sidak's multiple comparisons	Male: *p* = 0.0412, female: *p* = 0.9986
Extended Data Fig. 1-3*E*	Non-normal distribution	Two-way ANOVA, Sidak's multiple comparisons	Male: *p* = 0.8936, female: *p* = 0.9142
Extended Data Fig. 1-3*F*	Non-normal distribution	Two-way ANOVA, Sidak's multiple comparisons	Male: *p* = 0.9886, female: *p* = 0.3146
			
Fig. 2*A*	Non-normal distribution	Kruskal–Wallis, Dunn's multiple comparisons	ACC: Layer I: MIA+/CSH– *p* < 0.05, Layer II/III MIA+/CSH+ *p* < 0.05, PL: Layer I: MIA+/CSH– *p* < 0.05, Layer II/III MIA+/CSH+ *p* < 0.05
Fig. 2*B*	Non-normal distribution	Kruskal–Wallis, Dunn's multiple comparisons	ACC: Layer II/III: MIA+/CSH– *p* < 0.05, MIA–/CSH+ *p* < 0.05, MIA+/CSH+ *p* < 0.05, PL: Layer II/III: MIA+/CSH+ *p* < 0.05
Fig. 2*C*	Non-normal distribution	Kruskal–Wallis, Dunn's multiple comparisons	ACC: Layer I: MIA+/CSH– *p* < 0.05, MIA+/CSH+ *p* < 0.05, Layer II/III: MIA+/CSH+ *p* < 0.05, Layer V: MIA+/CSH+ *p* < 0.05, PL: Layer I: MIA+/CSH+ *p* < 0.05, Layer II/III: MIA+/CSH+ *p* < 0.05, Layer V: MIA+/CSH– *p* < 0.05
Fig. 2*D*	Non-normal distribution	Kruskal–Wallis, Dunn's multiple comparisons	ACC: Layer I: MIA+/CSH+ *p* < 0.05, Layer II/III: MIA+/CSH+ *p* < 0.05, PL: Layer I: MIA+/CSH– *p* < 0.05, MIA+/CSH+ *p* < 0.05, Layer II/III: MIA+/CSH– *p* < 0.05, MIA+/CSH+ *p* < 0.05, MIA+/CSH+ *p* < 0.05
Extended Data Fig. 2-1*A*	Non-normal distribution	Kruskal–Wallis, Dunn's multiple comparisons	ACC: Layer II/III: MIA+/CSH+ *p* < 0.05, PL: Layer II/III MIA+/CSH+ *p* < 0.05
Extended Data Fig. 2-1*B*	Non-normal distribution	Kruskal–Wallis, Dunn's multiple comparisons	ACC: Layer V MIA+/CSH– *p* < 0.05, MIA+/CSH+ *p* < 0.05, PL: Layer II/III: MIA+/CSH– *p* < 0.05, MIA+/CSH+ *p* < 0.05, Layer V: MIA+/CSH+ *p* < 0.05
Extended Data Fig. 2-1*C*	Non-normal distribution	Kruskal–Wallis, Dunn's multiple comparisons	ACC: Layer V MIA–/CSH+ *p* < 0.05, MIA+/CSH+ *p* < 0.05, PL: Layer V MIA–/CSH+ *p* < 0.05, MIA+/CSH+, Layer V I: MIA–/CSH+ *p* < 0.05
Extended Data Fig. 2-1*D*	Non-normal distribution	Kruskal–Wallis, Dunn's multiple comparisons	ACC: Layer V and Layer VI: MIA+/CSH– *p* < 0.05, MIA–/CSH+ *p* < 0.05, MIA+/CSH+ *p* < 0.05, PL: Layer II/III: MIA+/CSH– *p* < 0.05, MIA–/CSH+ *p* < 0.05, MIA+/CSH+ *p* < 0.05, Layer V: MIA+/CSH+ *p* < 0.05
Extended Data Fig. 2-1*D*	Non-normal distribution	Kruskal–Wallis, Dunn's multiple comparisons	ACC: Layer I: MIA–/CSH+ *p* < 0.05, PL: ns
			
Fig. 3*A*	Non-normal distribution	Two-way ANOVA, Sidak's multiple comparisons	MZ: *p* < 0.05, CP: *p* ≤ 0.05, SVZ/VZ *p* < 0.05
Fig. 3*B*	Non-normal distribution	Mann–Whitney	gad65+: *p* = 0.3333, ki67+: *p* = 0.0381, gad65+/ki67+: *p* = 0.0095
Fig. 3*C*	Non-normal distribution	Mann–Whitney	gad65+: *p* = 0.0012, brdu+: *p* < 0.001, gad65+/brdu+: *p* = 0.0012
Extended Data Fig. 3-1*A*	Non-normal distribution	Mann–Whitney	ns
Extended Data Fig. 3-1*B*	Non-normal distribution	Mann–Whitney	ns
Extended Data Fig. 3-1*C*	Non-normal distribution	Two-way ANOVA, Sidak's multiple comparisons	MZ: *p* = 0.714, CP: *p* = 0.04521, SVZ/VZ: *p* = 0.1657
Extended Data Fig. 3-2	Non-normal distribution	Mann–Whitney	*p* = 0.0688
Extended Data Fig. 3-3*B*	Non-normal distribution	Mann–Whitney	*p* = 0.0047
Extended Data Fig. 3-3*C*	Non-normal distribution	Mann–Whitney	*p* = 0.0315
			
Fig. 4*A*	Non-normal distribution	Kruskal–Wallis, Dunn's multiple comparisons	gad65+: MIA+/CSH+ *p* < 0.05;
Fig. 4*B*	Non-normal distribution	Kruskal–Wallis, Dunn's multiple comparisons	gad65+: MIA+/CSH+ *p* < 0.05; gad65+/brdu+: MIA+/CSH+ *p* < 0.05

Fig. 5	Non-normal distribution	Two-way ANOVA, Tukey's multiple comparisons	e17.5: MIA+/CSH– *p* < 0.001, P10: MIA+/CSH+ *p* < 0.05, P30: MIA+/CSH+ *p* < 0.05
			
Fig. 6*A*	Non-normal distribution	Mann–Whitney	iba1+: *p* = 0.0067, cd68+: *p* = 0.0163
Fig. 6*B*	Non-normal distribution	Kruskal–Wallis, Dunn's multiple comparisons	iba1+: ns, cd68+: MIA+/CSH+ *p* < 0.01
Fig. 6*C*	Non-normal distribution	Kruskal–Wallis, Dunn's multiple comparisons	iba1+: ns, cd68+: ns
			
Fig. 7*A*	Normal distribution	One-way ANOVA, Holm–Sidak's multiple comparisons	MIA+/CSH– *p* < 0.05, MIA+/CSH+ *p* < 0.001
Fig. 7*B*	Normal distribution	One-way ANOVA, Holm–Sidak's multiple comparisons	MIA+/CSH+ *p* < 0.01
Fig. 7*C*	Normal distribution	Two-way ANOVA, Tukey's multiple comparisons	ns
Fig. 7*D*	Normal distribution	Two-way ANOVA, Tukey's multiple comparisons	d2: MIA+/CSH+ *p* < 0.05, d3: MIA+/CSH+ *p* < 0.05
Fig. 7*E*	Normal distribution	One-way ANOVA, Holm–Sidak's multiple comparisons	ns
Fig. 7*F*	Normal distribution	One-way ANOVA, Holm–Sidak's multiple comparisons	MIA+/CSH+ *p* < 0.05
Fig. 7*G*	Normal distribution	One-way ANOVA, Holm–Sidak's multiple comparisons	MIA+/CSH– *p* < 0.05, MIA+/CSH+ *p* < 0.05
Extended Data Fig. 7-1*A*	Normal distribution	One-way ANOVA, Holm–Sidak's multiple comparisons	ns
Extended Data Fig. 7-1*B*	Normal distribution	One-way ANOVA, Holm–Sidak's multiple comparisons	ns
Extended Data Fig. 7-1*C*	Normal distribution	One-way ANOVA, Holm–Sidak's multiple comparisons	ns
Extended Data Fig. 7-1*D*	Normal distribution	One-way ANOVA, Holm–Sidak's multiple comparisons	ns
Extended Data Fig. 7-1*E*	Non-normal distribution	Kruskal–Wallis, Dunn's multiple comparisons	ns
Extended Data Fig. 7-1*F*	Normal distribution	One-way ANOVA, Holm–Sidak's multiple comparisons	ns

CP, cortical plate; MIA–/CSH–, mice treated with saline and reared under normoxia; MIA+/CSH-; mice subjected to MIA and reared under normoxia; MIA–/CSH+, mice treated with saline and reared under CSH; (MIA+/CSH+), mice subjected to MIA and reared under CSH; MZ, marginal zone; ns, non-significant; SVZ/VZ, subventricular/ventricular zone.

**Figure 1.**
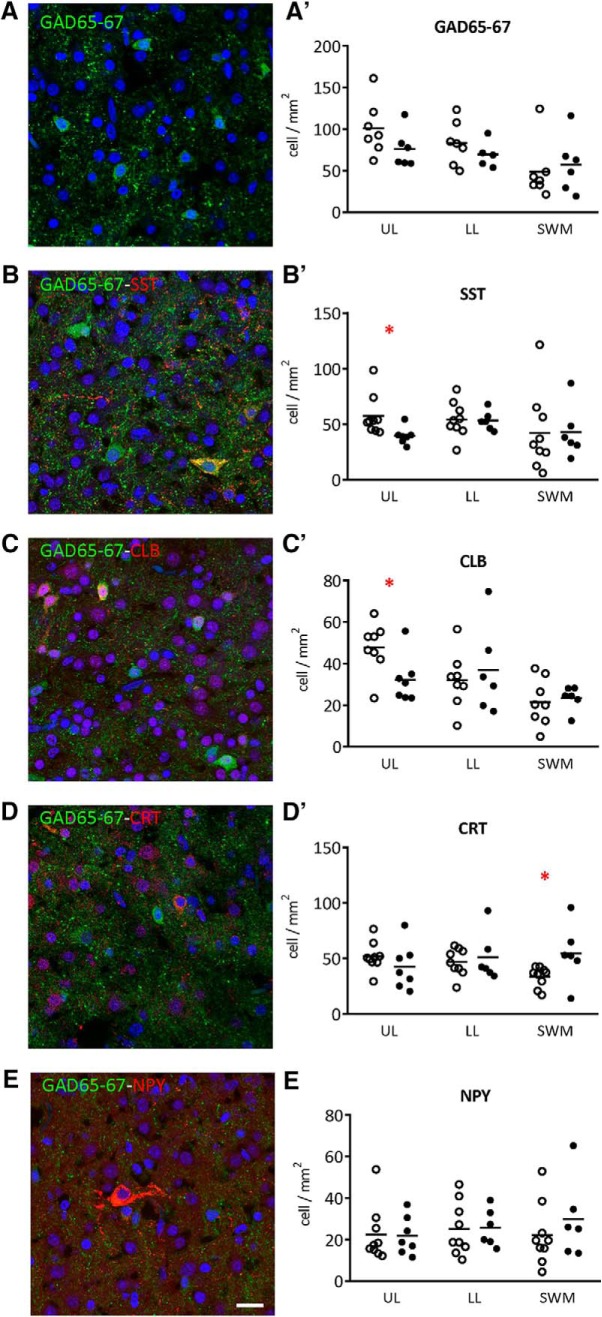
Effect of prematurity on interneurons density in BA9 of male infants. Illustrations of (***A***) glutamate decarboxylase 65 and 67 (GAD65-67, green), (***B***) SST (red), (***C***) CLB (red), (***D***) CRT (red), and (***E***) NPY (red) interneurons density. Scale bar = 20 μm. Quantification of (***A’***) GAD65-67, (***B’***) SST, (***C’***) CLB, (***D’***) CRT, and (***E’***) NPY positive cells in the upper layers (ULs), lower layers (LLs) and subcortical white matter (SWM) of the BA9 of the frontal cortex of term and preterm male infants. BA9 of female infants are presented on Extended Data Figure 1-1. Interneuron-related protein expression and transcripts are presented in Extended Data Figures 1-2, 1-3, respectively. Scatter dot plots represent the mean and individual dispersion of seven to nine term (empty circles) and six to seven preterm infants (full circles); *p < 0.05 (two-way ANOVAs were performed followed by Fisher’s LSD tests for *post hoc* comparisons).

10.1523/ENEURO.0300-18.2019.f1-1 Extended Data Figure 1-1 Effect of prematurity on interneurons density in BA9 of female infants. Quantification of (***A***) GAD65-67, (***B***) SST, (***C***) CLB, (***D***) CRT, and (***E***) NPY positive cells in the ULs, LLs, and SWM of the BA9 of the frontal cortex of term and preterm female infants. Scatter dot plots represent the mean and individual dispersion of three term (empty circles) and four preterm infants (full circles; two-way ANOVAs were performed followed by Fisher’s LSD tests for *post hoc* comparisons). Download Figure 1-1, TIF file.

**Figure 2.**
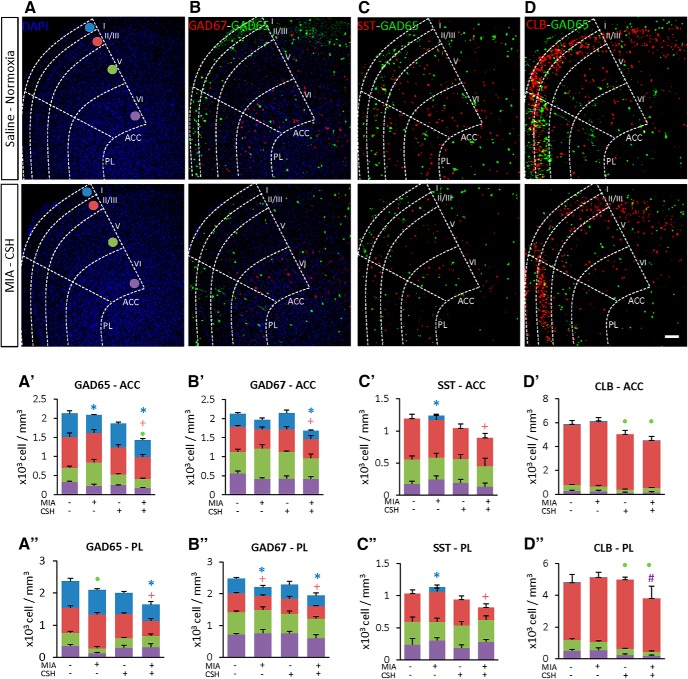
Effect of the multi-hit model on interneuron abundance and distribution in the anterior cingulate cortex (ACC) and prelimbic area (PL) of the PFC at P30. Illustration of (***A***) cortical layer delineation with DAPI (blue), the density and laminar distribution of GAD65 (green) overlaid with (***B***) GAD67, (***C***) SST, and (***D***) CLB (red) in the ACC and PL Layers I, II/III, V, and VI of mice treated with saline and reared under normoxia or subjected to MIA (injected with 150 μg/kg of LPS at E15.5 and E16.5) and reared under CSH. Scale bar = 100 μm. Five additional subtypes of interneuron were analyzed and are presented in Extended Data Figure 2-1. Quantification of interneurons (***A’–D’***) in the ACC and (***A’’–D’’***) in the PL of mice treated with saline and reared under normoxia, subjected to with MIA and reared under normoxia, treated with saline and reared under CSH and subjected to MIA and reared under CSH. Layers densities are stacked and blue, red, green, and purple colors are assigned for Layers I, II/III, V, and VI, respectively. Values represent the mean (±SEM) from five to six animals out two pregnancies. *, +, •, #*p* < 0.05 (Kruskal–Wallis with Dunn’s comparisons).

10.1523/ENEURO.0300-18.2019.f1-2 Extended Data Figure 1-2 Effect of prematurity on interneuron-related protein expression in BA9 of male and female term and preterm infants. ***A***, Representative blots of term (T) and preterm infants (PT). Quantification of protein expression of (***B***) glutamate decarboxylase 65 (GAD65), (***C***) GAD67, (***D***) SST, (***E***) CLB, and (***F***) CRT in term (T, white bars) and preterm infants (PT, black bars). GAPDH was used for normalization (two-way ANOVA with Sidak’s multiple comparisons). Download Figure 1-2, TIF file.

**Figure 3.**
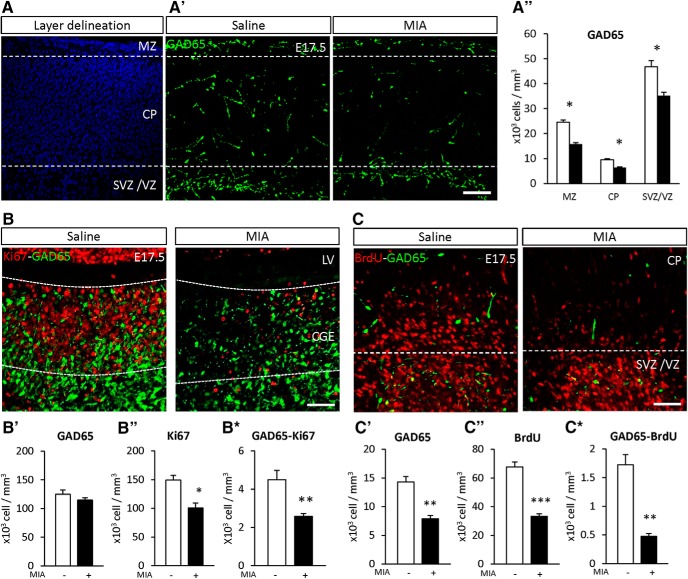
Effect of MIA at E17.5 on interneuron progenitor’s proliferation and fate. ***A***, Delineation of the sub-regions of the embryonic cortex with DAPI (blue). ***A’***, GAD65 density at E17.5 in the marginal zone (MZ), cortical plate (CP), and subventricular/ventricular zone (SVZ/VZ; green). Scale bar = 100 μm. ***A’’***, Quantification of GAD65 positive cells in the MZ, CP, SVZ/VZ. ***B***, GAD65 proliferation at E17.5 in the remaining CGE [density of GAD65 (green) and Ki67 (red)]. Scale bar = 50 μm. Proliferation in the other ganglionic eminence areas is presented in Extended Data Figure 3-1. Quantification of (***B’***) GAD65, **(*B’’***) Ki67, and (B*) GAD65 and Ki67 co-labeled cells density in the CGE; (***C***) GAD65 cell fate in the cerebral cortex at E17.5 [GAD65 (green) and BrdU (red)] in mice subjected to saline or MIA (injected with 150 μg/kg of LPS at E15.5 and E16.5). Scale bar = 50 μm. Quantification of (***C’***) GAD65, (***C’’***) BrdU, and (C*) GAD65 and BrdU positive cells in the cerebral cortex of mice treated with saline (white bars) and subjected to MIA (black bars). Apoptotic cell death and total neuronal densities are presented in Extended Data Figures 3-2, 3-3, respectively. Values represent the mean (±SEM) from five to six animals out two pregnancies. ***A’***, *p < 0.05 (two-way ANOVA with Sidak’s multiple comparisons); ***B***, ***C***, *p < 0.5, **p < 0.01, ***p < 001 (Mann–Whitney).

10.1523/ENEURO.0300-18.2019.f1-3 Extended Data Figure 1-3 Effect of prematurity on interneuron-related transcript expression in BA9 of male and female term and preterm infants. Quantification of mRNA levels of (***A***) *gad1*, (***B***) *gad2*, (***C***) *sst*, (***D***) *calb1*, (***E***) *calb2*, and (***H***) *npy* in term (T, white bars) and preterm infants (PT, black bars). *tfrc* was used for normalization; *p < 0.05 (two-way ANOVA with Sidak’s multiple comparisons). Download Figure 1-3, TIF file.

10.1523/ENEURO.0300-18.2019.f2-1 Extended Data Figure 2-1 Effect of the multi-hit model on interneuron abundance and distribution in the ACC and PL of the PFC at P30. Illustration of the density and laminar distribution of A-E, GAD65 (green) and overlaid with (***A***) PV, (***B***) VIP, (***C***) CRT, (***D***) NPY, and (***E***) RLN (red) in the ACC and PL Layers I, II/III, V, and VI of mice treated with saline and reared under normoxia or subjected to MIA (injected with 150 μg/kg of LPS at E15.5 and E16.5) and reared under CSH. Scale bar = 100 μm. Quantification of interneurons (***A’–E’***) in the ACC and (***A’’–E’’***) in the PL of mice treated with saline and reared under normoxia, subjected to with MIA and reared under normoxia, treated with saline and reared under CSH and subjected to MIA and reared under CSH. Layers densities are stacked and blue, red, green, and purple colors are assigned for Layers I, II/III, V, and VI, respectively. Values represent the mean (±SEM) from at five to six animals out of two pregnancies; *, +, •, #*p* < 0.05 (Kruskal–Wallis with Dunn’s comparisons). Download Figure 2-1, TIF file.

10.1523/ENEURO.0300-18.2019.f3-1 Extended Data Figure 3-1 Effect of MIA at E17.5 on Nkx2.1 interneurons progenitors. Illustration of Nkx2.1 density (***A***) in the medial ganglionic eminence (MGE), (***B***) in the preoptic area (POA), and (***C***) in the embryonic cortex, i.e., marginal zone (MZ), cortical plate (CP), and subventricular/ventricular zone (SVZ/VZ; green) of mice E17.5 subjected to saline or MIA (injected with 150 μg/kg of LPS at E15.5 and E16.5). Scale bar = 100 μm. Quantification of nk2.1-positive cells (***A****’*) in the MGE, (***B****’*) POA, and (***C****’*) cortex MZ, CP, SVZ/VZ positive cells in the cortex of mice treated with saline (white bars) and subjected to MIA (black bars). Values represent the mean (±SEM) from five to six animals out of two pregnancies. ***A’***, ***B’***, Mann–Whitney; ***C***’, *p < 0.05 (two-way ANOVA with Sidak’s multiple comparisons). Download Figure 3-1, TIF file.

10.1523/ENEURO.0300-18.2019.f3-2 Extended Data Figure 3-2 Effect of MIA at E17.5 on apoptotic cell death. ***A***, Illustration of cleaved-caspase 3 density, in the embryonic cortex of mice E17.5 subjected to saline or MIA (injected with 150 μg/kg of LPS at E15.5 and E16.5). Arrowheads highlight caspase 3 positive cells. The marginal zone (MZ), cortical plate (CP), and subventricular/ventricular zone (SVZ/VZ) are added for reference. Scale bar = 50 μm. ***B***, Quantification of cleaved-caspase 3 positive cells in the cortex of E17.5 mice treated with saline (white bars) and subjected to MIA (black bars). Values represent the mean (±SEM) from five to six animals out two pregnancies; *p < 0.05; ***p < 0.001 (Mann–Whitney). Download Figure 3-2, TIF file.

10.1523/ENEURO.0300-18.2019.f3-3 Extended Data Figure 3-3 Effect of MIA at E17.5 on cell density. ***A***, Illustration of neuronal (with NeuN) and cellular (with DAPI) density, in the embryonic cortex of mice E17.5 subjected to saline or MIA (injected with 150 μg/kg of LPS at E15.5 and E16.5). The marginal zone (MZ), cortical plate (CP), and subventricular/ventricular zone (SVZ/VZ) are added for reference. Scale bar = 50 μm. Quantification of (***B***) NeuN and (***C***) DAPI positive cells in the cortex of E17.5 mice treated with saline (white bars) and subjected to MIA (black bars). Values represent the mean (±SEM) from five to six animals out of two pregnancies; **p < 0.01 (Mann–Whitney). Download Figure 3-3, TIF file.

10.1523/ENEURO.0300-18.2019.f7-1 Extended Data Figure 7-1 Complementary behavioral characterization. Assessment of activity (***A***) in the Y-maze with the number of arm entries, (***B***) in the Barnes maze with the number of nose pokes, and (***C***) in the open field with the total distance traveled. Anxiety measurement in the open field: (***D***) time spent in the border of the arena, (***E***) time spent in the center of the arena, (***F***) count of number of buried marbles in the marble burying test of P30 mice reared under normoxia, subjected to with MIA (injected with 150 μg/kg of LPS at E15.5 and E16.5) and reared under normoxia, treated with saline and reared under CSH, subjected to MIA and reared under CSH. Values represent the mean (±SEM) from eight to twelve animals out of two pregnancies. ***A–D***, ***F***, non-significant (one-way ANOVA with Holm–Sidak’s multiple comparisons); ***E***, non-significant (Kruskal–Wallis test with Dunn’s multiple comparisons). Download Figure 7-1, TIF file.

## Results

### Major subtypes of interneurons are decreased in the human preterm PFC

The PFC, including BA9, is one of the brain regions most frequently altered in psychiatric disease. To address whether interneuron density in this region was altered by prematurity, BA9 sections from 13 term and 14 preterm infants were obtained. Mean age of death (absolute age) was 1.5 months in term infants and 3.7 months for preterm infants delivered between 26 and 34 weeks of gestation (average corrected age 1.1 months). Sex, gestational weeks and cause of death varied, but none were attributed to CNS infection, hemorrhage or malformation (Table 1). Genetic diseases or anatomic birth defects were excluded.

Interneuron subtypes express specific molecular markers, in addition to expressing markers for GABA synthesis (GAD65 and GAD67). These subtypes have distinct morphologies, connectivity and physiology that allow precise inhibitory control of local neural networks (Tremblay et al., 2016). Some types can be identified by non-overlapping marker expression [SST, PV, and sets of vasoactive intestinal peptide (VIP) expressing and non-expressing interneurons] while other molecular markers are expressed in populations that overlap with the major markers [CRT, CLB, or neuropeptide Y (NPY)]. Major subclasses of interneurons were assessed by immunostaining for GAD65-67, SST, CLB, CRT, and NPY to detect the major types of human interneurons for which antibodies are available [in male (Fig. 1) and female (Extended Data Fig. 1-1), term and preterm infants] not). Interneurons were counted in three sub-regions of BA9; the ULs, LLs, and the SWM.

A trend toward decrease was observed in the BA9 of human preterm male infants for GAD65-67^+^ (UL –24% ns, LL –16% ns, SWM +17%, ns; Fig. 1*A*,*A’*) and a significant reduction in the number of SST^+^ (UL –31% *p* < 0.05, LL –2% ns, SWM +1%, ns; Fig. 1*B*,*B’*) and CLB^+^ (UL –28% *p* < 0.05, LL +15% ns, SWM +9%, ns; Fig. 1*C*,*C’*) interneurons in upper but not LLs. CRT^+^ interneurons density was increased in the SWM (UL –17% ns, LL +9% ns, SWM +63%, *p* < 0.05; Fig. 1*D*,*D’*). No change was observed in the number of NPY^+^ interneurons (Fig. 1*E*,*E’*). PV^+^ cells were not detected in this set of human samples, although these cells could be detected at later developmental stages (data not shown). No statistically significant change in total cortical layer widths was discernable based on Nissl staining (data not shown), suggesting overall preservation of pyramidal cells at this age. No difference was observed in female infants, but statistical power was limited by the small sample size (Extended Data Fig. 1-1). Global interneuron-related protein expression was not altered in the BA9 of preterm infants (Extended Data Fig. 1-2); however, interneuron-related transcripts for *gad2* and *calb1* were increased (Extended Data Fig. 1-3).

### The multi-hit model alters interneurons density and distribution

To develop a new preclinical model that captures the multiple insults of human preterm brain injury, as well as the specific interneuron loss observed in the human samples studied here, prenatal MIA (induced by low dose LPS given late in gestation) was combined with postnatal CSH (induced by housing in a hypoxic environment for a week). Four experimental groups were used: mice whose dams were treated with saline and reared under normoxia (MIA–/CSH–); mice whose dams were treated with LPS and reared under normoxia (MIA+/CSH–); those treated with saline and reared in hypoxia (MIA–/CSH+); and those treated with LPS and reared in hypoxia (MIA+/CSH+; the “multi-hit” model).

Total interneuron density, illustrated in Figure 2*A–D*, was analyzed by counting cells positive for GAD65 and GAD67 in the two main sub-regions of the PFC, the ACC (Fig. 2*A’*,*B’*) and PL (Fig. 2*A’’*,*B’’*). Density and laminar distribution were analyzed and both GAD65^+^ [–36% *p* < 0.05 (Fig. 2*A’*); –33% *p* < 0.05 (Fig. 2*A’’*)] and GAD67^+^ [–22% *p* < 0.05 (Fig. 2*B’*); –23% *p* < 0.05 (Fig. 2*B’’*)] were significantly decreased in the MIA+/CSH+ cohort at P30. The multi-hit model also induced a long-lasting decrease in the density of SST^+^ [–32% *p* < 0.05 (Fig. 2*C’*); –57% *p* < 0.05 (Fig. 2*C’’*)] and CLB^+^ [–22% *p* < 0.05 (Fig. 2*D’*); –57% *p* < 0.05 (Fig. 2*D’’*)], similar to the findings in human preterm PFC. In this animal model, no interneuron sex differences were detected so sexes were combined in further analyses.

Five additional subtypes of interneuron present in the PFC of mice were analyzed: PV (Extended Data Fig. 2-1*A*), VIP (Extended Data Fig. 2-1*B*), CRT (Extended Data Fig. 2-1*C*), NPY (Extended Data Fig. 2-1*D*), and RLN (Extended Data Fig. 2-1*E*). All subtypes except RLN were reduced. Only PV^+^ [–32% *p* < 0.05 (Extended Data Fig. 2-1*A’*); –60% *p* < 0.05 (Extended Data Fig. 2-1*A’’*)] and SST^+^ (Fig. 2*C–C’’*), both in Layer II/III, were exclusively decreased after both hits. Similarly, MIA altered the density and distribution of VIP^+^, in ACC Layer V (MIA+/CSH– –70% *p* < 0.05; MIA+/CSH+ –31% *p* < 0.05; Extended Data Fig. 2-1*B’*) and PL Layer II/III (MIA+/CSH– –43% *p* < 0.05, MIA+/CSH+ –41% *p* < 0.05; Extended Data Fig. 2-1*B’’*). CSH alone led to a reduction in CLB^+^ in Layer V that was similar to the multi-hit model [MIA–/CSH+ –42% *p* < 0.05, MIA+/CSH+ –22% *p* < 0.05 (Fig. 2*D’*); MIA–/CSH+ –38% *p* < 0.05, MIA+/CSH+ –57% *p* < 0.05 (Fig. 2*D’’*)]. In contrast CRT^+^ and NPY^+^ subtypes were decreased after either MIA or CSH, as well as by the combination of insults (Extended Data Fig. 2-1*C*,*D*). Taken together, these data point out unique changes induced in the multi-hit model in GAD65, GAD67, PV, and SST expressing interneurons.

### PFC interneuron loss due to MIA-induced alteration of proliferation and migration plus CSH-induced maturation delay

To understand the mechanisms underlying the alterations induced by the multi-hit model in the PFC, the direct effect of mild late inflammation was examined at E17.5. Comparing fetuses from MIA versus saline exposed gestations revealed a significant decrease in the number of GAD65-GFP^+^ in three major sub-divisions of the developing cerebral cortex (MZ –36%, CP –33%, SVZ/VZ –25% *p* < 0.05; Fig. 3*A*–*A’’*). To elucidate the cause of interneuron loss, Ki67 (a marker of mitosis) was used in combination with GAD65 to label interneuron progenitors proliferation in the caudal ganglionic eminence (CGE), one of the three germinative areas where the majority of GAD65^+^ interneurons are generated. At E17.5, MIA significantly reduced Ki67^+^, and cells double-labeled for GAD65^+^ and Ki67^+^ [–32% *p* < 0.05 (Fig. 3*B’* *’*) –43% *p* < 0.01 (Fig. 3B*)]. Interneuron density in the medial ganglionic eminence (MGE) and preoptic area was determined by Nkx2.1 expression, a marker of interneuron progenitors. Like GAD65^+^, Nkx2.1^+^ cell density did not differ in these germinative zones but was significantly decreased in embryonic cortex (–35% *p* < 0.05; Extended Data Fig. 3-1).

To assess the contribution of survival and migration to the loss of cortical interneurons, MIA and saline control dams were injected at E15.5 with BrdU, which labels actively dividing cells during a restricted period (Fig. 3*C*). At E17.5, MIA exposure significantly decreased BrdU^+^ GAD65^+^ double-labeled cells in the PFC (–72% *p* < 0.01; Fig. 3C*). The contribution of apoptotic cell death to the loss of cortical interneurons was examined, but no significant difference was observed in cleaved caspase 3-positive cells (Extended Data Fig. 3-2). A small decrease in neuronal density was observed using NeuN^+^ (Extended Data Fig. 3-3), but this reduction was significantly less than the percentage of GAD65^+^ neurons lost. Overall, these data highlight alterations of interneuron proliferation and suggest an alteration of migration induced by MIA.

To examine the molecular factors involved in interneuron loss, the expression of 18 interneuron-related transcripts was assessed at E17.5 in the cerebral cortex of mice subjected to MIA or saline. A significant increase was observed in the expression of fate determination (*gad2*, *+*57%; *nkx2.1*, *+*142%; *lhx6*, *+*92%; *ki67*, *+*62%; Table 3) and migration mRNAs (*dlx1*, *+*51%; *dlx5*, *+*176%; Table 3) suggesting that early interneuron suppression may lead to a compensatory increases in genes that can promote subsequent interneuron progenitor production.

**Table 3.** Effect of MIA at E17.5 on the regulation of interneurons fate determination and migration-related transcripts

Gene symbol	Gene name	Fold change	SEM	Significance
gad1 (gad67)	Glutamate decarboxylase 1	1.11	0.07	
gad2 (gad65)	Glutamate decarboxylase 2	1.57	0.07	*
nkx2.1	NK2 homeobox 1	2.42	0.29	***
ascl1 (mash1)	Achaete-scute family bHLH transcription factor 1	1.39	0.09	
pax6	Paired box 6	1.02	0.16	
lhx6	LIM homeobox protein 6	1.92	0.19	*
ki67	Antigen identified by monoclonal antibody Ki67	1.62	0.08	*
dlx1	Distal-less homeobox 1	1.51	0.07	*
dlx2	Distal-less homeobox 2	1.12	0.17	
dlx5	Distal-less homeobox 5	2.76	0.53	***
dlx6	Distal-less homeobox 6	0.98	0.10	

Quantification of mRNA levels in E17.5 embryos. Each value represents the mean (±SEM) from at least five embryos out of at least two pregnancies; *p < 0.05; ***p < 0.001 (Mann–Whitney).

**Figure 4.**
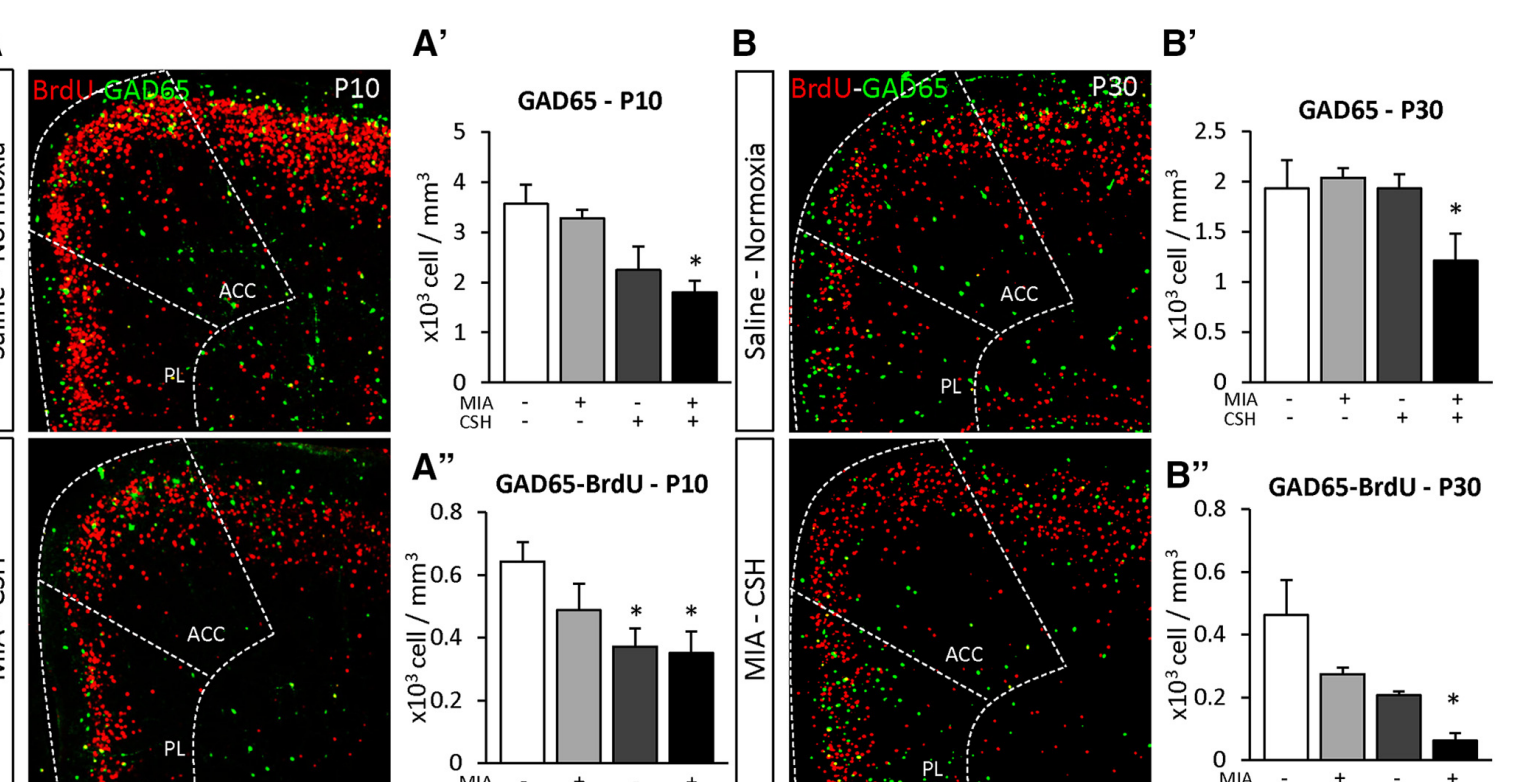
Effect of the multi-hit model on interneuron density and fate in the PFC at (***A***) P10 and (***B***) P30. GAD65 cell fate in the PFC [GAD65 (green) and BrdU (red)] of (***A***) mice treated with saline and reared under normoxia or subjected MIA and reared under CSH. Scale bar = 100 μm. Quantification of (***A’***) GAD65, (***B’***) GAD65 and BrdU co-labeled cell density of P10; and (***A’’***) GAD65, (***B’’***) GAD65 and BrdU co-labeled cell density at P30 in the PFC (ACC and PL are added for reference) treated with saline and reared under normoxia, subjected to with MIA and reared under normoxia, treated with saline and reared under CSH, subjected to MIA and reared under CSH. Values represent the mean (±SEM) from five to six animals out two pregnancies; *p < 0.05 (Kruskal–Wallis test with Dunn’s multiple comparisons).

**Figure 5.**
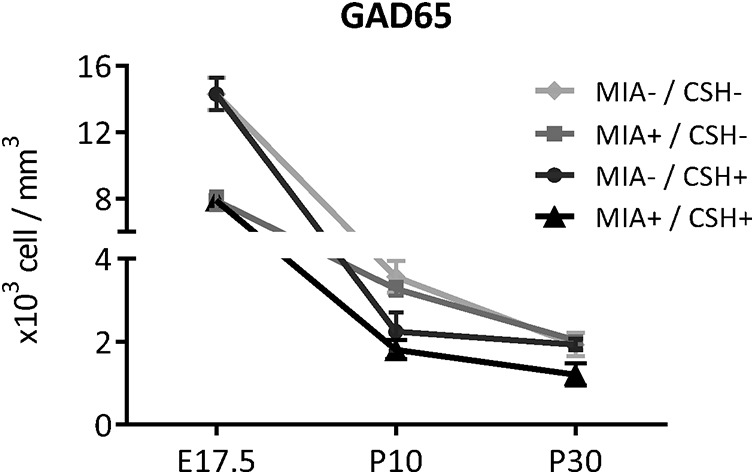
Effect of the multi-hit model on GAD65 positive cells density at E17.5, P10, and P30 in the PFC of mice treated with saline and reared under normoxia, subjected to with MIA and reared under normoxia, treated with saline and reared under CSH, subjected to MIA and reared under CSH. Values represent the mean (±SEM) from at least seven animals out of at least two pregnancies; *p < 0.05; ***p < 0.001 (two-way ANOVA, Tukey’s multiple comparisons).

The addition of postnatal hypoxia was then assessed (Fig. 4). In MIA+/CSH– mice, GAD65^+^ cell numbers recovered by P10. This recovery was abolished by addition of CSH (MIA+/CSH– –8% ns, MIA+/CSH+ –49% *p* < 0.05; Fig. 4*A’*). With hypoxia treatment alone (MIA–/CSH+), fewer GAD65^+^ cells were also seen, suggesting a direct effect of hypoxia on interneuron survival which was confirmed by co-labeling GAD65 with BrdU (injected at E15.5; Fig. 4*A’’*). MIA+/CSH+ cortex at P30 showed long-lasting interneuron deficits (Fig. 4*B–B’’*). Overall, MIA induced an early loss of interneurons through proliferation and migration defects which, with the addition of CSH, prevented recovery so that these deficits persisted into adulthood (MIA+/CSH+ E17.5% –44% *p* < 0.001, P10 –49% *p* < 0.05, P30 –37% *p* < 0.05; Fig. 5).

### The addition of CSH induced a second burst of inflammation

**Figure 6.**
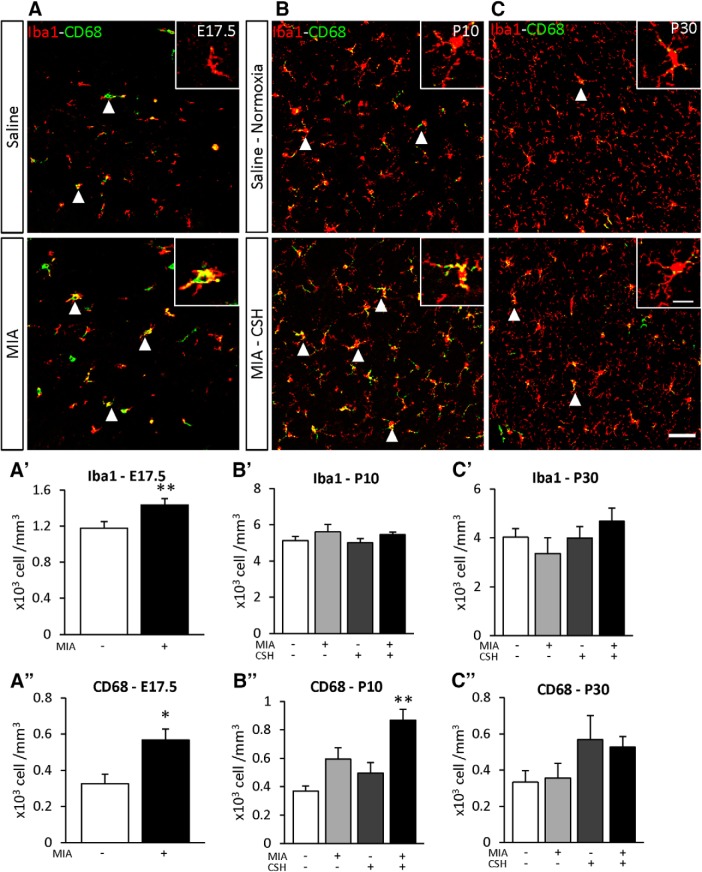
Effect of the multi-hit model on microglial density (Iba1) and activation (CD68) in the PFC at (***A***) E17.5 subjected to saline or MIA (injected with 150 μg/kg of LPS at E15.5 and E16.5), (***B***) P10, and (***C***) P30 mice treated with saline and reared under normoxia or subjected to MIA and reared under CSH. Arrowheads highlight cells positive for Iba1 and CD68. Scale bar = 50 μm. Scale bar of inset = 15 μm. Quantification of Iba1 at (***A’***) E17.5, (***B’***) P10, (***C’***) P30 and CD68, (***A’’***) E17.5, (***B’’***) P10, (***C’’*)** P30 positive cells of mice treated with saline and reared under normoxia, subjected to with MIA and reared under normoxia, treated with saline and reared under CSH, subjected to MIA and reared under CSH. Values represent the mean (±SEM) from five to six animals out two pregnancies. ***A’***, ***A’’***, *p < 0.05; **p < 0.01 (Mann–Whitney); ***B’–C’’***, **p < 0.01 (Kruskal–Wallis test with Dunn’s multiple comparisons).

To determine the effect of the multi-hit model on inflammation, the density of Iba1 positive cells was used to determine the total number of microglia and Iba1^+^ CD68^+^ double-labeled cells detected microglial activation. At E17.5, microglial numbers (+22% *p* < 0.05; Fig. 6*A’*) and microglial activation (+75% *p* < 0.01; Fig. 6*A’’*) were increased by MIA. By P10, only MIA+/CSH+ significantly increased activated microglia (MIA+/CSH– +61%, ns; MIA–/CSH+ +34%, ns; MIA+/CSH+ +135%, *p* < 0.01; Fig. 6*B’’*), suggesting a sensitization of microglial cells by MIA to a second inflammatory response induced by hypoxia. These inflammatory markers had declined by P30 although interneuron loss persisted (Fig. 6*C–C’’*).

### The multi-hit model alters working memory, cognitive flexibility, and social cognition

To further define long-term behavioral alterations potentially linked to PFC GABAergic network deficits, neurobehavioral tests for working memory, cognitive flexibility and social cognition were performed.

**Figure 7.**
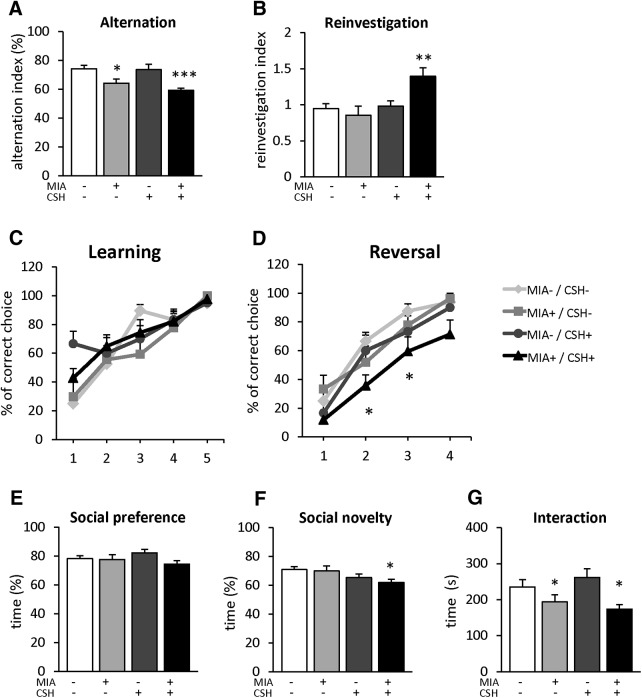
Behavioral characterization. Assessment of working memory with spontaneous alternation in the Y-maze, mice were allowed to freely explore the maze for 10 min. ***A***, Alternation index. Reinvestigation in the Barnes maze, mice were allowed to freely explore the maze for 10 min. ***B***, Reinvestigation index. Assessment of learning and reversal learning in the water T-maze. Percentage of correct choice during (***C***) the learning phase (5 d) and (***D***) the reversal phase of the test (4 d). Assessment of social cognition in the three-chamber test. Percentage of time spent interacting between (***E***) a conspecific stranger (S1) versus an object; (***F***) a novel (S2) versus a familiar (S1) conspecific; (***G***) total time spent to interacting with conspecifics strangers of P30 mice treated with saline and reared under normoxia, subjected to with MIA (injected with 150 μg/kg of LPS at E15.5 and E16.5) and reared under normoxia, treated with saline and reared under CSH, subjected to MIA and reared under CSH. Complementary information is presented in Extended Data Figure 7-1. Values represent the mean (±SEM) from eight to twelve animals out of two pregnancies. ***A***, ***B***, ***E–G***, *p < 0.05; **p < 0.01; ***p < 0.001 (one-way ANOVA with Holm–Sidak’s multiple comparisons); ***C***, ***D***, *p < 0.05; **p < 0.01; ***p < 0.001 (two-way ANOVA with Tukey’s multiple comparisons).

Working memory was analyzed through the spontaneous alternation task in the Y-maze, where the alternation level was significantly lower in mice exposed to MIA+/CSH+, and similar in mice exposed to MIA only (MIA+/CSH– –13%, ns; MIA+/CSH+ –20% *p* < 0.001; Fig. 7*A*). This working memory deficit was confirmed in the Barnes maze test where the repeated investigation of previously explored holes was significantly higher in MIA+/CSH+ animals (+47% *p* < 0.01; Fig. 7*B*), while the number of arm entries and nose-pokes remained stable across groups (Extended Data Fig. 7-1*A*,*B*).

Cognitive flexibility was examined using the reversal-learning task of the water T-maze. No difference was observed for learning the initial location of the platform (Fig. 7*C*). In contrast, during the reversal learning phase, MIA+/CSH+ mice required more time to learn the new location, with lower performance on days 2 and 3 (day 2, –35%, *p* < 0.05; day 3, –59%, *p* < 0.05; Fig. 7*D*). No significant difference was observed between groups by day 4 (Fig. 7*D*).

Social cognition was evaluated with social preference and novelty in the three-chamber test. During social preference, all the groups spent >70% of the time with the conspecific animal (Fig. 7*E*). A similar pattern was observed during social novelty testing, where groups spent more time with the unfamiliar mouse (Fig. 7*F*). However, although MIA+/CSH+ mice discriminate between the unfamiliar and familiar animal, they spent significantly less time interacting with the unfamiliar animal compared to controls (–12% *p* < 0.05; Fig. 7*F*) and exhibited decreased total interaction time (–26% *p* < 0.05; Fig. 7*G*). No significant difference was observed for activity and anxiety in the open-field (Extended Data Fig. 7-1*C–E*) or for repetitive behavior with the marble burying test (Extended Data Fig. 7-1*F*).

Overall, this new multi-hit mouse model decreases several subtypes of interneurons in the ULs of the PFC and induces behavioral deficits characteristic of schizophrenia-like disorders. The observation of a pronounced decrease in the density of multiple interneuron subtypes in the human preterm brain samples examined here suggests that alterations of interneurons following extreme prematurity might be a risk factor for psychiatric disorders or other neurodevelopmental disabilities that can follow preterm birth.

## Discussion

Elucidating the effects of perinatal insults on GABAergic interneuron development is critical to understanding the mechanistic role they play in the pathogenesis of neuropsychiatric disorders. While human glutamatergic neurogenesis is complete by 28 weeks of gestation (Malik et al., 2013), GABAergic progenitors persist in the ganglionic eminences through at least 35 weeks of gestation and continue to migrate into the cortex throughout the perinatal period (Sanai et al., 2011; Arshad et al., 2016; Paredes et al., 2016), suggesting that perinatal insults may be significant environmental factors predisposing preterm survivors to psychiatric disorders.

In the present study, the BA9 of one-month-old term and one-month term corrected-age very preterm infants were compared. A decrease in the density of both SST and CLB interneuron subtypes was seen in the ULs of the frontal cortex from these preterm infants. Human pathology studies remain limited, but our findings are consistent with prior studies showing altered interneuron numbers in preterm infants (aged from a few hours to a few weeks postpartum) in the white matter and subplate (Robinson et al., 2006), and in the cortical plate (Panda et al., 2018).

The human tissue used here comes from preterm donors who survived up to five months and were subjected to the adverse environment of prematurity for a significant period. While the insults that lead directly to death may have contributed to our findings, the immediate cause of death was similar in both the preterm and term infants (most commonly “sudden infant death syndrome,” while confounding genetic or obviously infectious diagnoses were excluded). However, the possibility remains that the observed PFC interneuron deficit may be linked not only to chorioamnionitis and respiratory compromise but to a variety of perinatal insults, many of which remain undefined. Human data suggest that generation of interneurons continues well into the postnatal period (Yang et al., 2011; Arshad et al., 2016; Paredes et al., 2016) raising the possibility that human survivors of extreme prematurity could subsequently produce interneurons to compensate for the observed loss. Many preterm infants survive neurologically intact and this postmortem sampling may reflect a more compromised population or a delay in interneuron development that would have recovered. In the future, non-invasive methods that measure localized GABA concentrations in living infants may help clarify these issues, but such techniques do not yet have the resolution required, so models must be used to better understand the progression of injury and underlying mechanisms.

Pairing two previously established methods (MIA and CSH) created a novel model of preterm encephalopathy that more closely mimics this human pathology. Unlike other multi-hit models (Jantzie et al., 2014; Mallard and Vexler, 2015), the use of MIA and CSH spans both pre- and postnatal periods, which is more representative of when preterm encephalopathy develops. Preclinical models of preterm brain injury are most successful when using multifactorial approaches that account for the complexity of the perinatal environment (Elovitz and Mrinalini, 2004; Manuel et al., 2017). The multi-hit mouse model was designed to investigate the effects of perinatal insults associated with preterm birth, producing a model in which later neurobehavioral disorders could be assessed.

Density of both GAD65 and GAD67 expressing cells was reduced, consistent with prior reports of prematurity-related decreases in *gad1* and *gad2* transcripts, encoding GAD67 and GAD65, in the PFC (Richetto et al., 2014; Labouesse et al., 2015). The decrease observed here was specific to Layer II/III. In mice, cortical interneurons are generated in the MGE between E9.5 and E15.5 with a peak at E12.5, contributing to the cortical layers in an inside-out manner (Butt et al., 2007), and from the CGE between E12.5 and E18.5, with a peak at E16.5, contributing to the superficial layers (Miyoshi et al., 2007, 2010; Torigoe et al., 2016). This data suggests that MIA altered the proliferation and migration of interneurons from the late MGE or from the CGE. A prior transcriptomic study using MIA shown down-regulation of transcripts involved in interneuron tangential migration including the Distal-less (*Dlx*) family and both GAD isoforms (Oskvig et al., 2012), suggesting that impaired migration may also play a role in the findings observed in E17.5 cortex. Prenatal stress has also been shown to reduce the distribution of interneurons by impairing their migration (Stevens et al., 2013).

While PV neurons are known to be selectively altered by MIA (Canetta et al., 2016), the other interneuron subtypes have been less comprehensively studied in the context of perinatal brain injury. In mouse PFC, a reduced density and laminar distribution of PV is observed, as well as decreased numbers of SST, CLB, VIP, CRT, and NPY after combined MIA plus CSH. This is the first demonstration of decreased density of CLB, CRT, and NPY in a model of perinatal brain injury. A delay in maturation of these neurons may explain these effects, as suggested by a study in which hypoxia induced a decrease in PV, SST, and VIP immunoreactivity that partially recovered at later stages (Komitova et al., 2013). The data from the ganglionic eminences suggest decreased interneurogenesis following MIA, followed by possible maturation defects as a consequence of postnatal CSH, supporting a combination of alterations on interneuron development.

MIA produces an early alteration of interneurogenesis followed by possible maturation defects as a consequence of postnatal CSH, but the underlying mechanisms by which maternal inflammation or postnatal hypoxia perturbs brain development remain unknown. Blocking the actions of specific cytokines such as IL-1β prevents MIA-induced behavioral and physiologic consequences in mouse offspring, suggesting a role for pro-inflammatory cytokines in the process (Girard et al., 2010). In support of this, in the multi-hit model, a transient activation of microglial cells is showed after MIA, which is significantly enhanced by later CSH. These findings, along with a recent report in a different mixed model (Zhou et al., 2017), highlight the extension of MIA-induced inflammatory response by CSH, suggesting that postnatal insults potentiate abnormalities caused by *in utero* inflammation to permanently alter brain development.

To further define long-term behavioral alterations potentially linked to GABAergic network deficits in the PFC, a battery of neurobehavioral tests was performed. The multi-hit model of preterm brain injury induced deficits in working memory, cognitive flexibility and social interaction. Direct alterations of excitatory and inhibitory balance within the PFC have a strong effect on social motivation, predominantly mediated by PV and SST interneuron subtypes (Bicks et al., 2015), potentially underlying the deficits seen in social novelty and social interaction.

Alterations in working memory have been extensively discussed in the context of psychiatric disorders. MIA, a known cause of such disorders, has been shown to impair working memory in offspring (Murray et al., 2017). Additionally, specific lesion of PV neurons in the PFC produces deficits in working memory and cognitive flexibility (Murray et al., 2015). Involvement of SST interneurons has also been demonstrated, although PV and SST interneurons show distinct contributions to PFC circuit dynamics underlying working memory (Kim et al., 2016). Impaired working memory in the multi-hit model may be associated with reduced GABAergic transmission by PV and SST interneurons in the adult PFC, but further investigations are needed to directly link specific interneuron losses with the observed behavioral phenotypes. Interestingly, lesion of the PFC or complete disruption of GABA_A_-receptor-mediated inhibition in the PFC by bicuculline infusion has been shown to recapitulate the behavioral deficits induced by the multi-hit model (Auger and Floresco, 2014; Hernan et al., 2014; Murray et al., 2015) supporting the hypothesis that the observed interneuron loss could cause the measured behavioral deficits.

In contrast to prior studies, here MIA alone did not impair working memory. This difference may stem from uses of lower LPS doses or timing of administration (Meyer et al., 2008). On the other hand, perinatal hypoxia alone induces impairments in associative learning, spatial memory, and long-term social memory that have been linked with white matter deficits (Cengiz et al., 2011; Lan et al., 2011; Salmaso et al., 2012). White matter injury has been mechanistically linked to interneuron deficits because interneurons promote oligodendrocytes development (Voronova et al., 2017), with hypoxic interneuron loss potentially contributing to impaired myelination.

Abnormalities in cortical interneurons have been broadly associated with cognitive deficits in the context of psychiatric disorders. The reduction of multiple interneuron subtypes in this model is consistent with prior observations in patients with schizophrenia. This pathology is also commonly cited with changes in GABA system-related transcripts, with altered expression of GABA-synthesizing enzymes (GAD65 and GAD67), GABA transporter systems and interneuron markers (SST, NPY, CLB, RLN, and cholecystokinin) reported (Hashimoto et al., 2003, 2008a,b; Maldonado-Avilés et al., 2009; Mellios et al., 2009; Volk et al., 2012). In this model, the potentiation of inflammation by postnatal CSH may increase the risk of psychiatric-related neurobehavioral deficits by producing latent inflammation with microglial activation across a critical developmental time (Meyer et al., 2011). Whether other more subtle indicators of latent inflammation persist into adulthood after the microglial activation subsides, as has been suggested for human autism spectrum disorders and schizophrenia (Meyer et al., 2011), remains to be explored. While the mechanistic links between loss of specific PFC interneurons and specific psychiatric deficits in both mouse and human need to be further investigated, the observed deficits are consistent with the cognitive (working memory and cognitive flexibility) and negative symptoms (social dysfunction) of schizophrenia. Thus, this model provides new opportunities to interrogate the molecular mechanisms that link perinatal insults, interneuron deficits and later neuropsychiatric risk.

## Synthesis

Reviewing Editor: Margaret McCarthy, Univ of Maryland School of Medicine Decisions are customarily a result of the Reviewing Editor and the peer reviewers coming together and discussing their recommendations until a consensus is reached. When revisions are invited, a fact-based synthesis statement explaining their decision and outlining what is needed to prepare a revision will be listed below. The following reviewer(s) agreed to reveal their identity: Alexandre Bonnin. Note: If this manuscript was transferred from JNeurosci and a decision was made to accept the manuscript without peer review, a brief statement to this effect will instead be what is listed below.

This study provides strong evidence that a mixed injury model (MIA in pregnancy and CSH in postnatal offspring) is a good tool to better understand the cellular alterations of specific sets of interneurons observed in preterm encephalopathy in humans. The data describing this animal model are very good. However there is concern regarding the human data. Comments are organized below into those relevant to the animal model and those relevant to the human data. The consensus is that additional data will be required for appropriate interpretation of the findings in premature infants. The authors can choose to either obtain that data, or remove all the human data from the manuscript and temper the conclusions accordingly. The consensus is this is a potentially very important new model and so including appropriate human data would be preferred as it would greatly increase the translational impact of the findings.

Comments on the animal model portion (in no particular order).

1. Space permitting, a few more details should be provided for the immunohistochemical procedures (e.g. buffers used, incubation times etc).

2. Methods specify that pups of both sexes were included in the study. For behavioral studies were males or females offspring (or both) used at P30? Given the male prevalence of mental diseases that this study is modelling (ASD, Schizophrenia), this consideration is potentially important.

3. Fig.3 A and C panel appear to show the same region of the E17.5 cortex (?), shown at similar magnification (identical scale bars). Yet, the number of GAD65+ cells in the CP appears quite different between panel A and C - therefore it is unclear 1) if the same region of the cortex is depicted (the legend only says “cerebral cortex”), or 2) if the magnification factor is different (higher magnification in panel C seems more likely based on the relative sizes of CP and SVZ). Also, legend should specify which region of the cerebral cortex is shown.

4. Fig. 6 legend should specify which region of the cerebral cortex is shown.

5. Determination of cortical layer divisions was based on apparent DAPI+ cell density, according to an earlier publication (Ref. Skorput et al 2015). In the referenced publication, the actual DAPI staining was shown in order to support the arbitrary divisions; similarly here, a few panels (maybe 1 per figure) showing DAPI staining should be presented in Figure 2-4 in order to justify ROI choices. This would clarify how the authors insured that layer delineations were identical in each section without using specific layer markers.

6. Introduction. Several sentences have awkward wording and should be written more clearly or carefully. Lines 50-53; “maturation into the frontal lobe”; awkward sentence. Lines 55-58; ‘interneuron alterations could contribute to the increased risk“; meaning needs to be clarified. By interneuron alterations do you mean alterations in interneuron numbers or connectivity or function, or all of these?

7. Introduction, last sentence. The sentence “This multi-hit protocol is proposed as a novel preclinical model to decipher the cellular and physiological mechanisms underlying the deficits in survivors of prematurity that may make them vulnerable to later neuropsychiatric disorders” implies vulnerability in all survivors of prematurity, rather than in the subset with inflammation and hypoxia. It should be qualified.

8. Methods, Maternal Immune Activation. More details are needed in this section. The strain of E. Coli that the LPS is from needs to be specified. Clarify if 150 μg/kg of LPS was given to the pregnant dam on both E15.5 and 16.5, or was 150 μg/kg the total dose with 75 μg/kg given each day? Specify how LPS was administered to the dam. Was i.p. injection used?

9. Methods, and Chronic Sublethal Hypoxia. More information is needed about the chronic sublethal hypoxia method. What effect does this have on the dam's milk production and treatment of pups? Is growth of the pups normal, or are the pups smaller as in the original paper using this method with 9.5% O2? Is this actually “intermittent” hypoxia? Are they normoxic when cages are changed? What is the flow rate of the gas? Is it sufficient to cool the pups? Is the gas mixture warmed prior to delivery? Are controls housed in a similar environment with 21% O2?

10. Methods, Mouse tissue. Specify how dams were euthanized to obtain the fetal brains. Specify for brain samples how many litters of pups were used and if pups for each treatment were from multiple litters. Specify when BrdU was administered to the mice where brains were studied at P30.

11. Discussion, line 397. Specify if “some data” refers to data from human infants or from animal studies.

12. Discussion, lines 426-437. The authors suggest that their data support a delay in maturation of several populations of GABAergic neurons. This is supported by the data. The question of whether this is a delay in “interneurogenesis” is less clear as delayed maturation of neuronal precursors may not always reflect less neurogenesis.

Comments on the human data

1. The comparison of data from brains of very premature infants to “age matched” term infants is a major concern as these groups would be very different in terms of brain development. Presumably “age matched” refers to postnatal age but this is not specified in the methods. If one calculates post-conception age (using 1 month =4 weeks), the term infants range from 56-60 weeks post conception age; whereas the very premature infants that ranged from 26-29 weeks gestation and were studied at 3-5 months postnatal age which would be 40-49 weeks post-conception age. Thus the very preterm infant population studied were equivalent to post-conception ages ranging from newborn (40 weeks pc) to 9 weeks old (49 weeks pc). A t test comparing the post conception ages calculated from the data in table 1 is very highly significant with a p value of 7.897E-06 (post conception age for term infants and very preterm infants are 57.7 +/- 2.1 and 45 +/- 3.6, weeks respectively). Thus the brain of the very preterm infants should be compared to samples from term infants from 40-49 weeks post conception (1-9 weeks of postnatal age). Comparing the data from very preterm infants to infants of a significantly older post-conception age is not a meaningful comparison.

2. The second major concern is the underlying assumption and implication that the data from 6 very preterm infants, 3 of which had conditions with hypoxia/anoxia is representative of all very preterm infants. This is not a valid assumption and findings from this very small sample should not be generalized to population of very premature infants.

3. Abstract and discussion, regarding the human brain data. The number of samples of human brain tissue are small (7 term infants, from 4-5 months of age and 6 preterm infants, from 3-5 months of age), and the samples from premature infants are from infants with identified or unidentified pathology. Three of the preterm infants had conditions associated with hypoxia/anoxia including pneumonia and asphyxia, and 3 premature infants died from SIDS or Sudden Unexplained Death in Infancy. The findings from the premature infants should be presented and discussed in this context. The concern is that the findings are presented and discussed as “premature brain”, with the implication that the samples and findings are representative of all premature infants. This reviewer recognizes the difficulty in obtaining samples from infant brain. However, the authors should more carefully phrase their interpretation and include caveats that the findings may not be representative of all premature brains. Thus, the sentence in lines 13-15 which states “In human PFC at 4 months of age, preterm birth is associated with a specific loss of somatostatin and calretinin interneurons in upper cortical layers” needs to be qualified as it is representative of premature brain from infants with hypoxia/anoxia and SIDS, not of healthy premature infants. Similarly, the sentence in lines 19-20 which states “The combined insults lead to long-term neurobehavioral deficits that parallel human psychopathologies seen after extremely preterm birth” should be qualified as it implies that the deficits and psychopathologies are seen after all (as opposed to just some) extremely premature births. The human data are relevant to a specific subset of premature infants, but are not necessarily relevant to all premature infants, many of whom have relatively normal neurodevelopmental outcomes.

4. Introduction, last sentence. The sentence “This multi-hit protocol is proposed as a novel preclinical model to decipher the cellular and physiological mechanisms underlying the deficits in survivors of prematurity that may make them vulnerable to later neuropsychiatric disorders” implies vulnerability in all survivors of prematurity, rather than in the subset with inflammation and hypoxia. It should be qualified.

5. Clarify if first paragraph of the results that 3 of 6 preterm infants had postnatal conditions with hypoxia or asphyxia. The amount of variability in the data from the preterm infants should be discussed.

6. Results; human brain data. Please clarify what “absolute age” means with regards to the very preterm infants in Table 1. Is this postnatal age? The numbers of infants are very small and considerable brain development takes place between 3 and 5 months of age. It is unfortunate that two of the premature infant brains were taken from such a young age. In addition, the question of whether the brains of very premature infants can validly be compared to the brains of term infants of the same postnatal age, or whether the premature brains should have been compared to infants of the same postconception age needs to be discussed as it is central to the interpretation of the data from the brains from the very premature infants.

7. Figure, specific concerns. Figure 1 NPY scatter plot. Clarify why data from only 4 of the 6 premature infants are shown in this figure. Figure 5 of results should have different colors or different types of lines as it is very difficult to identify the groups at E17.5.

8. Results. The last sentence is overstated. The wording “Since very preterm human samples also show a pronounced decrease in the density of multiple interneuron subtypes...” implies that brains from all very preterm infants would be similar to the samples studied from the 6 very preterm infants with hypoxia/asphyxia, SIDS and sudden unexplained death. This is not a valid assumption so the sentence should be qualified.

9. Discussion. There are many places in the discussion where the text implies that the data from the 6 infants from this study are representative of all very premature infants. This is not a valid assumption as some very preterm infants have no cognitive problems or neuropsychiatric disorders. Specific text that should be modified: Line 389 “following prematurity” is too global; it should be changed to be more specific wording e.g., “in the premature brains studied” or “in brains of premature infants with hypoxia and asphyxia conditions”.
