# Supplementary figures and images for: Impaired Interneuron Development in a Novel Model of Neonatal Brain Injury
Source: eNeuro. 2019 Feb 22;6(1):ENEURO.0300-18.2019. doi: 10.1523/ENEURO.0300-18.2019 (PMC6390196; doi:10.1523/ENEURO.0300-18.2019)

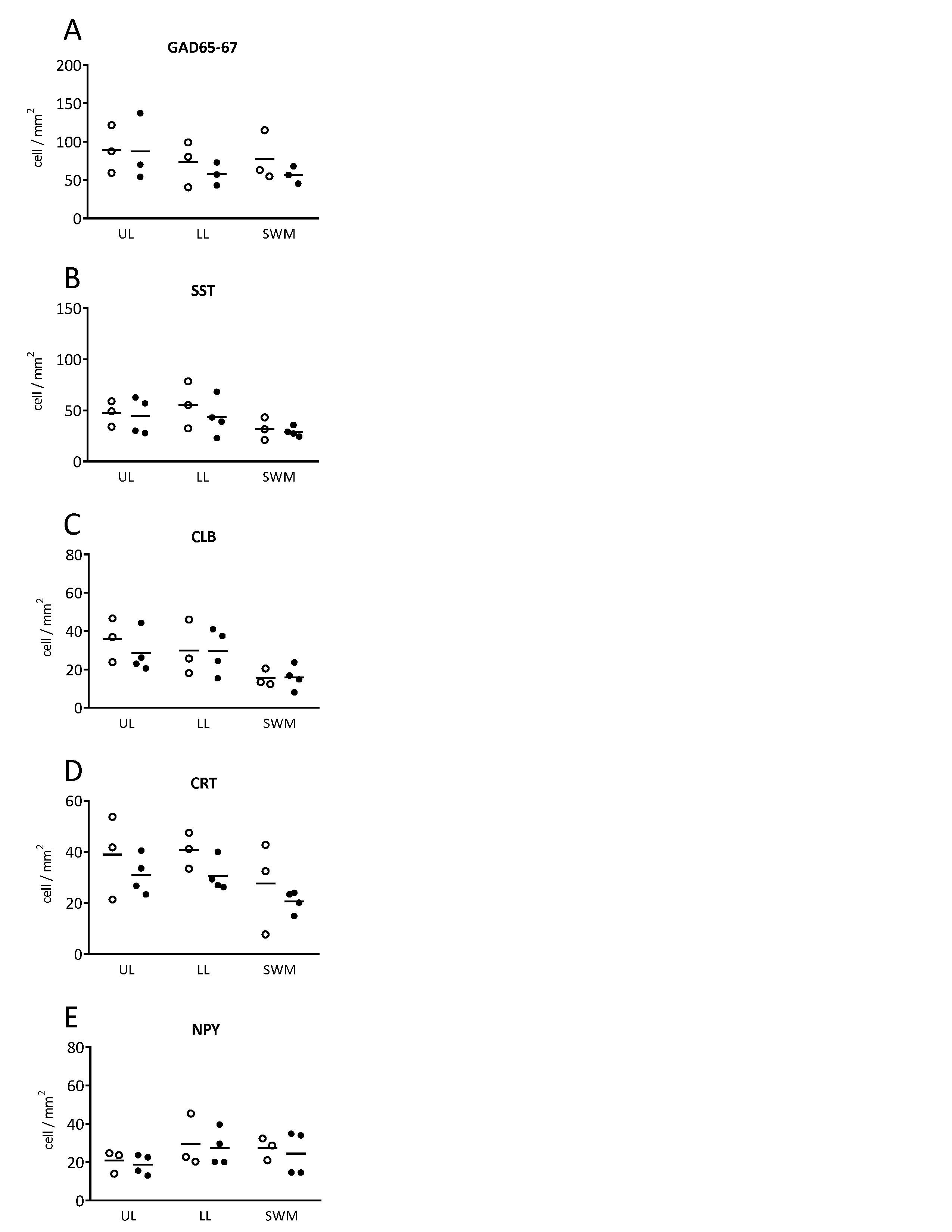

Supplement: Extended Data Figure 1-1 — Effect of prematurity on interneurons density in BA9 of female infants. Quantification of (A) GAD65-67, (B) SST, (C) CLB, (D) CRT, and (E) NPY positive cells in the ULs, LLs, and SWM of the BA9 of the frontal cortex of term and preterm female infants. Scatter dot plots represent the mean and individual dispersion of three term (empty circles) and four preterm infants (full circles; two-way ANOVAs were performed followed by Fisher’s LSD tests for post hoc comparisons). Download Figure 1-1, TIF file. [file sup_enu-eN-NWR-0300-18-s02.tif]

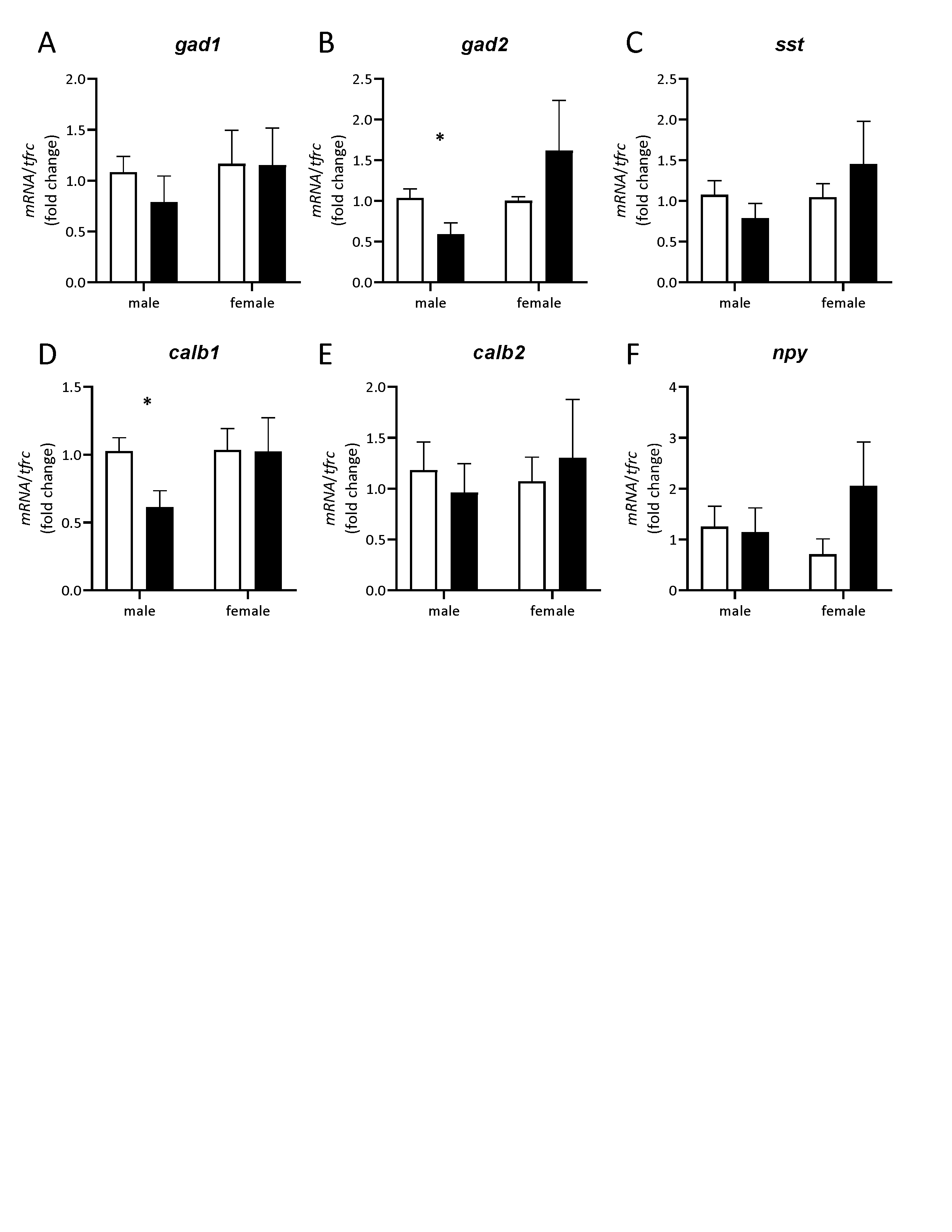

Supplement: Extended Data Figure 1-3 — Effect of prematurity on interneuron-related transcript expression in BA9 of male and female term and preterm infants. Quantification of mRNA levels of (A) gad1, (B) gad2, (C) sst, (D) calb1, (E) calb2, and (H) npy in term (T, white bars) and preterm infants (PT, black bars). tfrc was used for normalization; *p < 0.05 (two-way ANOVA with Sidak’s multiple comparisons). Download Figure 1-3, TIF file. [file sup_enu-eN-NWR-0300-18-s04.tif]

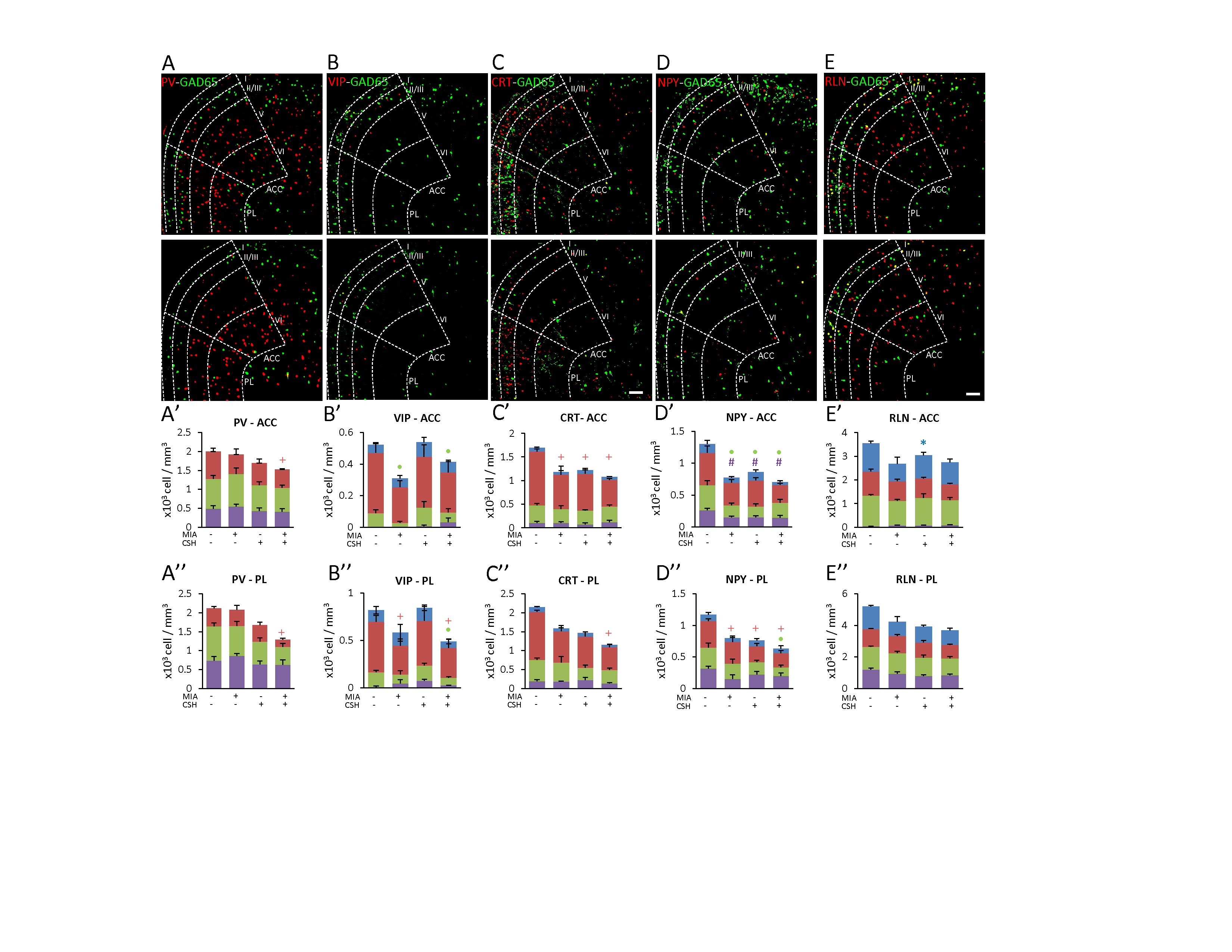

Supplement: Extended Data Figure 2-1 — Effect of the multi-hit model on interneuron abundance and distribution in the ACC and PL of the PFC at P30. Illustration of the density and laminar distribution of A-E, GAD65 (green) and overlaid with (A) PV, (B) VIP, (C) CRT, (D) NPY, and (E) RLN (red) in the ACC and PL Layers I, II/III, V, and VI of mice treated with saline and reared under normoxia or subjected to MIA (injected with 150 μg/kg of LPS at E15.5 and E16.5) and reared under CSH. Scale bar = 100 μm. Quantification of interneurons (A’–E’) in the ACC and (A’’–E’’) in the PL of mice treated with saline and reared under normoxia, subjected to with MIA and reared under normoxia, treated with saline and reared under CSH and subjected to MIA and reared under CSH. Layers densities are stacked and blue, red, green, and purple colors are assigned for Layers I, II/III, V, and VI, respectively. Values represent the mean (±SEM) from at five to six animals out of two pregnancies; *, +, •, #p < 0.05 (Kruskal–Wallis with Dunn’s comparisons). Download Figure 2-1, TIF file. [file sup_enu-eN-NWR-0300-18-s05.tif]

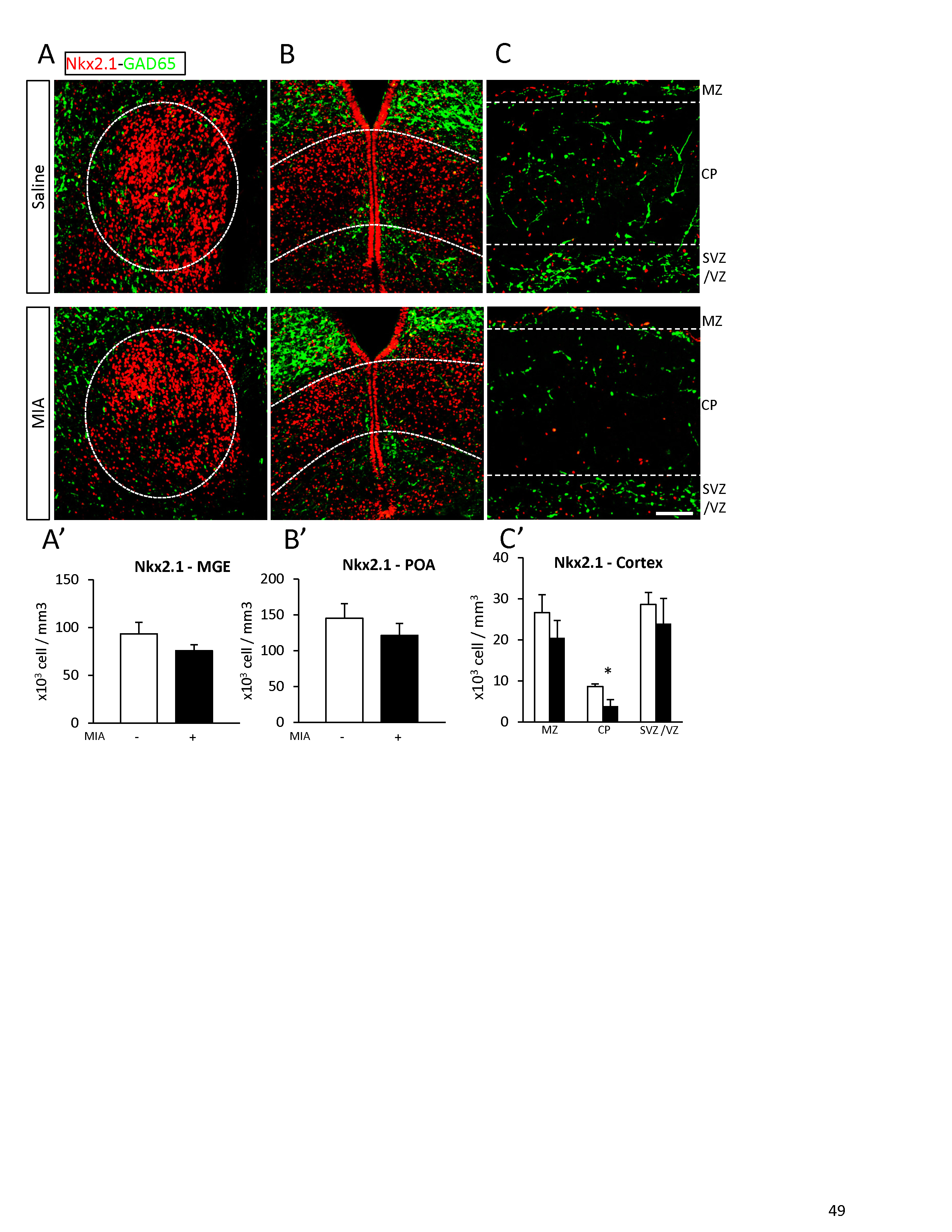

Supplement: Extended Data Figure 3-1 — Effect of MIA at E17.5 on Nkx2.1 interneurons progenitors. Illustration of Nkx2.1 density (A) in the medial ganglionic eminence (MGE), (B) in the preoptic area (POA), and (C) in the embryonic cortex, i.e., marginal zone (MZ), cortical plate (CP), and subventricular/ventricular zone (SVZ/VZ; green) of mice E17.5 subjected to saline or MIA (injected with 150 μg/kg of LPS at E15.5 and E16.5). Scale bar = 100 μm. Quantification of nk2.1-positive cells (A’) in the MGE, (B’) POA, and (C’) cortex MZ, CP, SVZ/VZ positive cells in the cortex of mice treated with saline (white bars) and subjected to MIA (black bars). Values represent the mean (±SEM) from five to six animals out of two pregnancies. A’, B’, Mann–Whitney; C’, *p < 0.05 (two-way ANOVA with Sidak’s multiple comparisons). Download Figure 3-1, TIF file. [file sup_enu-eN-NWR-0300-18-s06.tif]

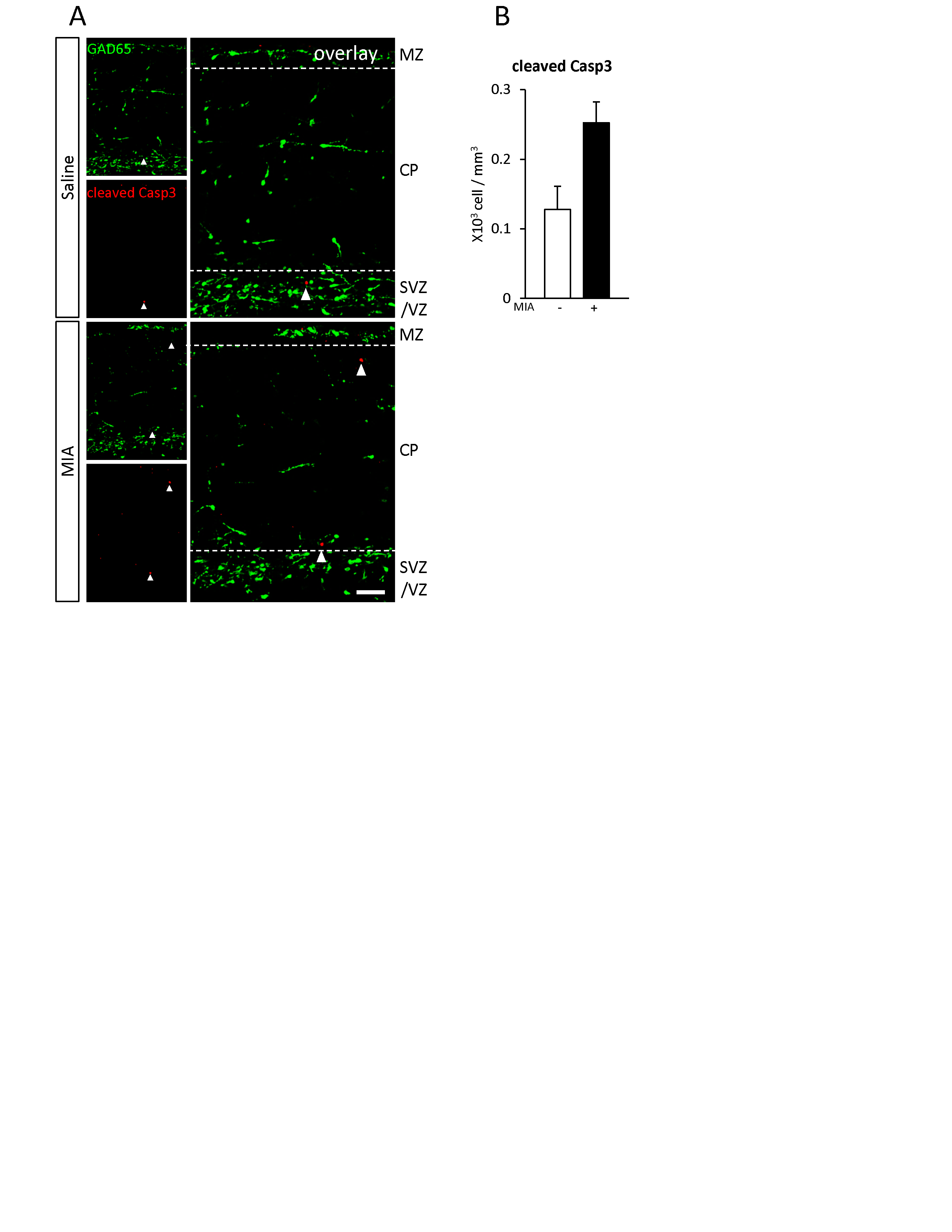

Supplement: Extended Data Figure 3-2 — Effect of MIA at E17.5 on apoptotic cell death. A, Illustration of cleaved-caspase 3 density, in the embryonic cortex of mice E17.5 subjected to saline or MIA (injected with 150 μg/kg of LPS at E15.5 and E16.5). Arrowheads highlight caspase 3 positive cells. The marginal zone (MZ), cortical plate (CP), and subventricular/ventricular zone (SVZ/VZ) are added for reference. Scale bar = 50 μm. B, Quantification of cleaved-caspase 3 positive cells in the cortex of E17.5 mice treated with saline (white bars) and subjected to MIA (black bars). Values represent the mean (±SEM) from five to six animals out two pregnancies; *p < 0.05; ***p < 0.001 (Mann–Whitney). Download Figure 3-2, TIF file. [file sup_enu-eN-NWR-0300-18-s07.tif]

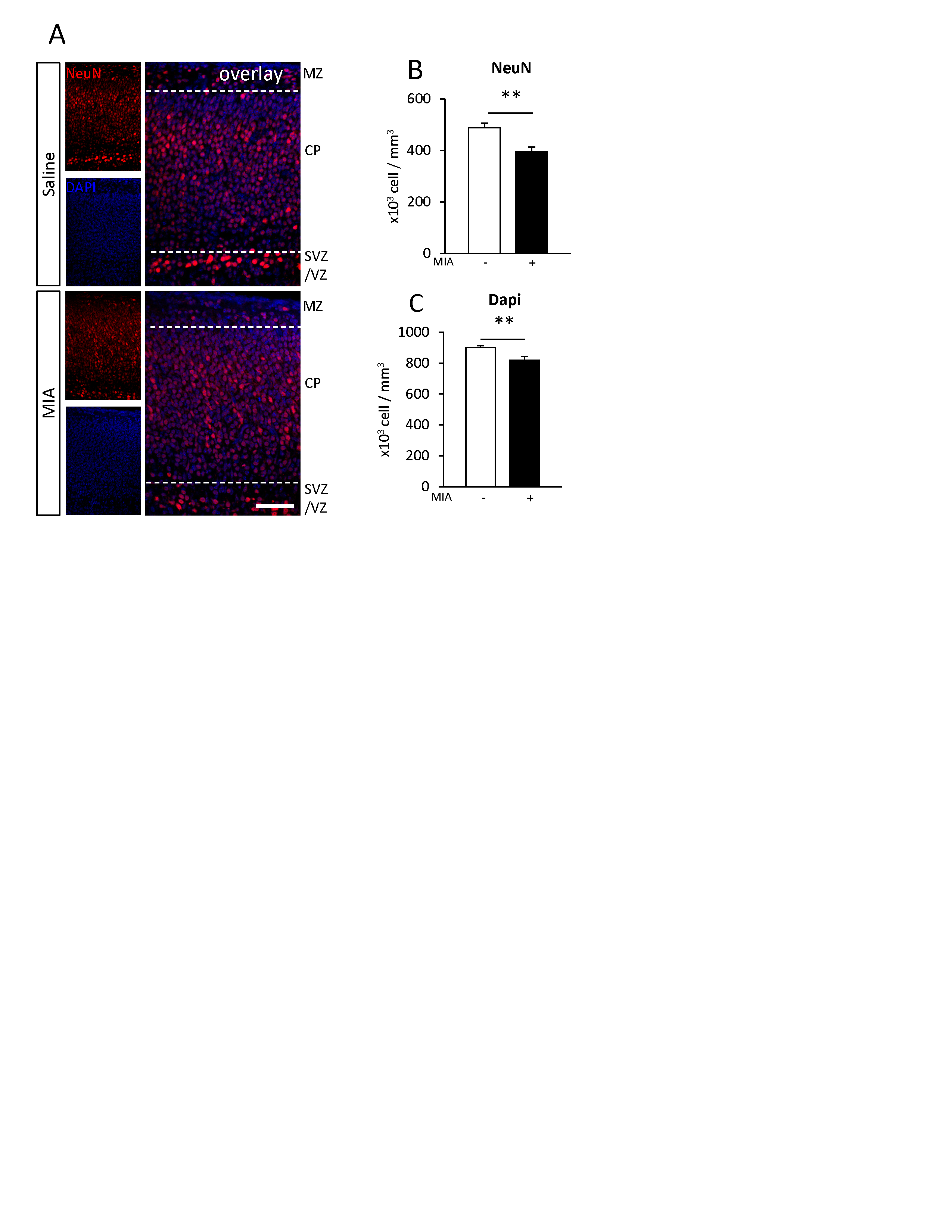

Supplement: Extended Data Figure 3-3 — Effect of MIA at E17.5 on cell density. A, Illustration of neuronal (with NeuN) and cellular (with DAPI) density, in the embryonic cortex of mice E17.5 subjected to saline or MIA (injected with 150 μg/kg of LPS at E15.5 and E16.5). The marginal zone (MZ), cortical plate (CP), and subventricular/ventricular zone (SVZ/VZ) are added for reference. Scale bar = 50 μm. Quantification of (B) NeuN and (C) DAPI positive cells in the cortex of E17.5 mice treated with saline (white bars) and subjected to MIA (black bars). Values represent the mean (±SEM) from five to six animals out of two pregnancies; **p < 0.01 (Mann–Whitney). Download Figure 3-3, TIF file. [file sup_enu-eN-NWR-0300-18-s08.tif]

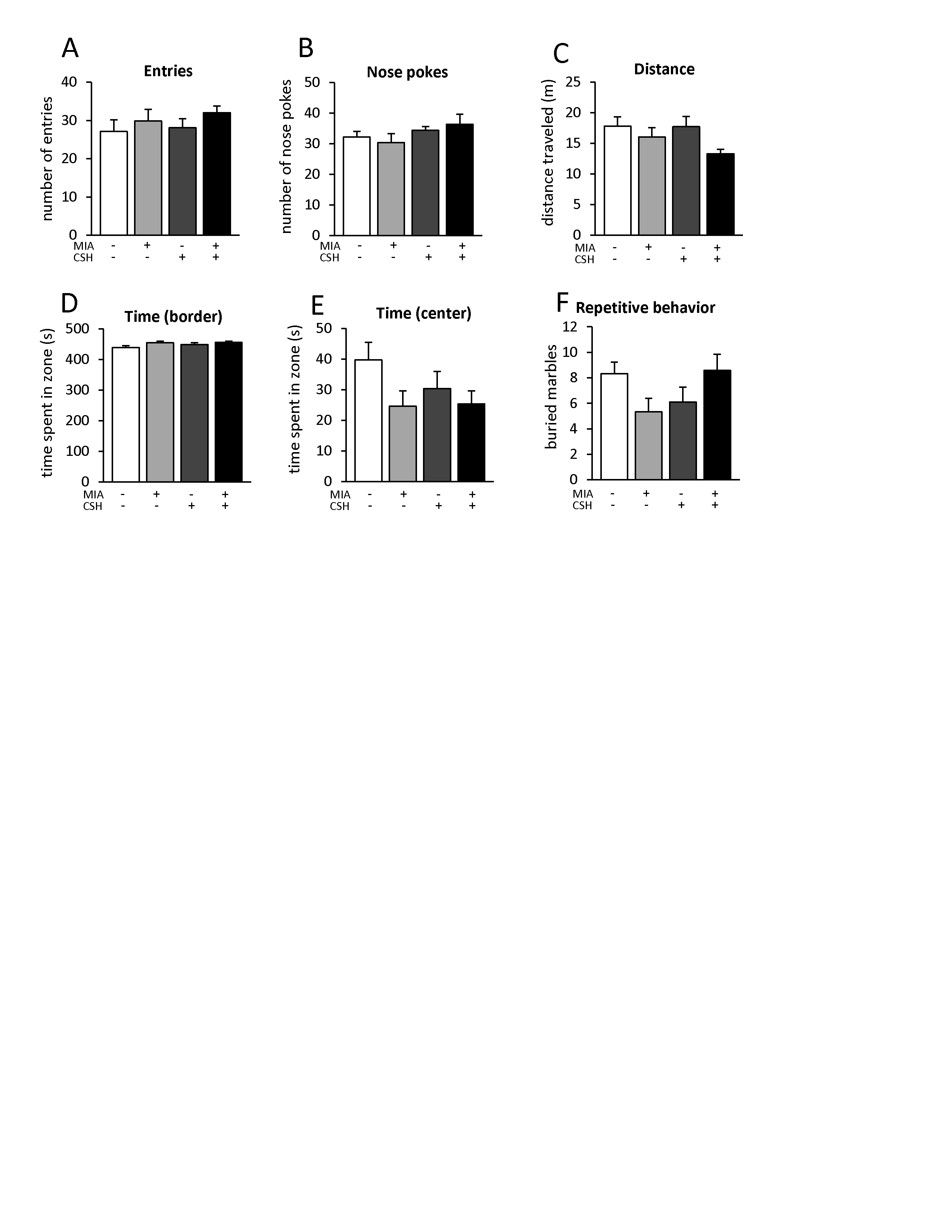

Supplement: Extended Data Figure 7-1 — Complementary behavioral characterization. Assessment of activity (A) in the Y-maze with the number of arm entries, (B) in the Barnes maze with the number of nose pokes, and (C) in the open field with the total distance traveled. Anxiety measurement in the open field: (D) time spent in the border of the arena, (E) time spent in the center of the arena, (F) count of number of buried marbles in the marble burying test of P30 mice reared under normoxia, subjected to with MIA (injected with 150 μg/kg of LPS at E15.5 and E16.5) and reared under normoxia, treated with saline and reared under CSH, subjected to MIA and reared under CSH. Values represent the mean (±SEM) from eight to twelve animals out of two pregnancies. A–D, F, non-significant (one-way ANOVA with Holm–Sidak’s multiple comparisons); E, non-significant (Kruskal–Wallis test with Dunn’s multiple comparisons). Download Figure 7-1, TIF file. [file sup_enu-eN-NWR-0300-18-s09.tif]
